# A scoping review of programmes that address the daily living functioning of people diagnosed with borderline personality disorder

**DOI:** 10.1177/00048674251393159

**Published:** 2025-11-29

**Authors:** Dillon Tepper, Ben Sellar, Sheryl Shipley, Rachel Smith, Carolyn M Murray

**Affiliations:** Occupational Therapy Program, Allied Health and Human Performance, University of South Australia, Adelaide, SA, Australia

**Keywords:** Psychiatric rehabilitation, mental health recovery, independent living skills, activities of daily living, clinical interventions

## Abstract

**Introduction:**

People diagnosed with borderline personality disorder experience persistent functional impairment despite current evidence-based treatment. Domains of daily living functioning, such as establishing a routine, household management and self-care, can be important goals for rehabilitation. This review aimed to scope and synthesise programmes that address the daily living functioning of people diagnosed with borderline personality disorder.

**Methods:**

This review followed the PRISMA Extension for Scoping Reviews Checklist and the Joanna Briggs Institute scoping review methodological guidance. Eight databases (Medline, PsycINFO, ASSIA, CINAHL, Embase, Emcare, ProQuest Dissertations & Theses Global and Scopus) and grey literature were searched.

**Results:**

Forty-four records were found spanning 12 countries, from 1987 to 2025, comprising research studies, educational materials and programme summaries. Programmes included independent living skills development, residential facilities, outpatient treatment and occupational therapy, with rehabilitation and recovery the most frequently used approaches. The domains of daily living functioning were health (*n* = 189), relational (*n* = 84), responsibility (*n* = 67), personal (*n* = 61), leisure (*n* = 53), routine (*n* = 42) and household (*n* = 30). Measures used to describe changes in functioning varied considerably.

**Conclusions:**

Programmes supporting the daily living functioning of people diagnosed with borderline personality disorder exist. However, the evidence base is currently disparate. No single programme addressed all identified domains of daily living functioning. Comprehensive, evidence-based rehabilitation programmes addressing all domains of daily living functioning are needed to enable functional recovery for people diagnosed with borderline personality disorder.

## Introduction

Borderline personality disorder (BPD) is a severe mental illness characterised by intense and unstable relationships, distorted sense of self, emotional dysregulation and impulsivity (American Psychiatric Association, 2013). Self-harm and suicidality are hallmark features (Lak et al., 2025). People diagnosed with BPD are prevalent in psychiatric settings (Zimmerman et al., 2005) and frequently seek support from emergency and mental health services during periods of crisis (Warrender et al., 2020).

BPD is complex and challenging to treat. Medications can manage some symptoms, but they do not lessen the severity of the disorder (Gartlehner et al., 2021; Stoffers-Winterling et al., 2022). Clinical practice guidelines currently recommend psychotherapy as the primary treatment for BPD (Aslam et al., 2025). However, only 50% of people who receive evidence-based psychotherapy respond to treatment (Woodbridge et al., 2022). Long-term functional impairment remains a significant problem (Álvarez-Tomás et al., 2019; Choi-Kain et al., 2020). Persistent challenges in functional recovery manifest as disability benefits (Kramer et al., 2023) and prolonged psychotherapy (Zanarini et al., 2004). From patient perspectives, increased support is needed to develop practical skills and participate more meaningfully in daily activities (Katsakou and Pistrang, 2018; Woodbridge et al., 2023).

Research into the functioning of people diagnosed with BPD currently focuses on symptomatic remission, having one emotionally sustaining relationship (relational functioning) and full-time work or study (vocational functioning) (Grenyer et al., 2022; Zanarini et al., 2010a, 2018). The complexity of functioning is reduced to a single score on the Global Assessment of Functioning (GAF) (Endicott et al., 1976; Gold, 2014). Relational and vocational domains have been synthesised elsewhere (Di Bartolomeo et al., 2024; Kernot et al., 2023; Lazarus et al., 2014; Sinnaeve et al., 2015; Steele et al., 2019). However, this work has not captured the multi-faceted nature of daily living functioning, and more attention to other domains is required. Daily living functioning refers to activities and routines in daily life, such as household management, financial responsibilities, leisure pursuits and self-care.

The literature that describes programmes addressing other domains of daily living functioning remains disparate, yet these programmes may provide important rehabilitation support and enable functional recovery. Therefore, the aim of this review was to scope and synthesise such programmes. The review question was, ‘*What programs are available to support people diagnosed with BPD to address their daily living functioning?*’

## Methods

This review followed Arksey and O’Malley’s (2005) framework for scoping reviews, the PRISMA Extension for Scoping Reviews (PRISMA-ScR) Checklist (Tricco et al., 2018) and the Joanna Briggs Institute (JBI) methodological guidance (Peters et al., 2020). The protocol was pre-registered on the Open Science Framework (https://osf.io/h87ea/).

### Inclusion criteria

The population for this review was ‘people diagnosed with BPD’, the concept was ‘daily living functioning’, and the context was ‘programmes’ (with no restrictions on settings). A BPD diagnosis was based on self-report, formal research methods or a health professional’s assessment (using *Diagnostic and Statistical Manual of Mental Disorders* [*DSM*] or *International Classification of Diseases* [*ICD*] frameworks). Records describing programmes addressing daily living functioning for people diagnosed with BPD aged 16+ were included. Programs could be structured and facilitator-led or self-management resources. Grey literature and relevant secondary research articles were also included, with the latter set aside for citation searching. There were no date or language restrictions in the database search, with Google Translate used for non-English records. Programmes based on established psychotherapies were included if they specifically addressed domains beyond relational or vocational functioning. All comorbidities were included, provided the programme involved at least one person diagnosed with BPD. Records that focused on mental illness without mention of BPD were excluded, as were programmes that only addressed relational or vocational functioning.

### Record sources

Eight databases were searched: Medline, PsycINFO, ASSIA, CINAHL, Embase, Emcare, ProQuest Dissertations & Theses Global and Scopus. For grey literature, Google and Google Scholar were searched alongside BPD organisation websites internationally. These organisations were also contacted directly to identify any additional relevant programmes.

### Search strategy

Search terms were developed with support from an academic librarian and informed by the domains of daily living functioning outlined by Desrosiers et al. (2017), including activities and routines in daily life such as self-care, health, household, financial, time management and leisure. Supplementary File 1 contains the complete search terms for all databases and the grey literature search strategy, with all searches conducted on the same day by the first reviewer. Medline was used to test and refine the final search strategy. Citation searching was conducted using the reference lists of included records and the ‘cited by’ function in Google Scholar was used to locate new records.

### Record screening and selection

Records retrieved from the search strategy on 5 August 2025 were initially uploaded into Endnote (Clarivate, 2024) and then transferred into Covidence (Veritas Health Innovation, 2024) for duplicate removal. Two reviewers independently screened titles and abstracts in Covidence, followed by full-text screening. An independent third reviewer resolved discrepancies.

### Data extraction and synthesis

Characteristics of the included records (author, year, country and aim), programme details (aim, approach, facilitator and setting) and measures used to describe changes in functioning were extracted into Excel. All extraction was completed by the first reviewer, with a second reviewer independently checking the extraction of 15% of the included records to ensure relevancy and accuracy. A third reviewer resolved disagreements. Domains of daily living functioning (health, relational, responsibility, personal, leisure, routine and household) were synthesised based on Desrosiers et al.’s (2017) assessment tool. The domains of daily living functioning addressed by programmes were coded using conventional content analysis (Hsieh and Shannon, 2005), using frequency counts of content. The measures used to describe changes in functioning within the quantitative studies were collated and critiqued for reliability and validity. The Excel worksheets were then converted into tables, with regular team discussions at all stages of the process.

## Results

**Figure 1.**
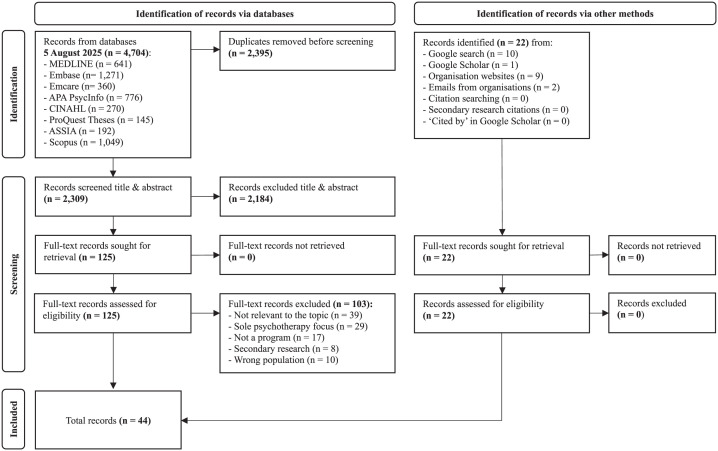
Flowchart of search and screening results.

A flowchart of the search and screening results is available in Figure 1. The database search identified 2,309 records for title and abstract screening, from which 2,184 were excluded. There were 125 full-text records, from which 22 were included. An additional 22 records were identified via other search methods, leading to 44 inclusions overall.

### Record characteristics

The characteristics of the 44 included records are outlined in Table 1. The records dated from 1987 and spanned across 12 countries, with the highest representation from Australia and New Zealand (Borderline in the ACT, 2025; Department of Health and Aged Care, 2024; Healthtalk Australia, 2025; Lee et al., 2014; National Disability Insurance Scheme [NDIS], 2025; Orygen, 2024; Project Air, 2018, 2020; Royal Australian & New Zealand College of Psychiatrists [RANZCP], 2017; Sane, 2022; Scanlan et al., 2017; Sekharan et al., 2021) and the United States (Brogan et al., 2020; Emotions Matter, 2020; John Hopkins Medicine, 2025; Life Adjustment Team, 2025; McAlpine, 1991; McLean Hospital, 2024; Mehl, 1997; National Alliance on Mental Illness [NAMI], 2017; Nelson and Condrin, 1987; Pacific Teen Treatment, 2024; Tomasi et al., 2019; verywell mind, 2023), with 12 records each, followed by the United Kingdom (Clark et al., 2006; Lamont et al., 2017; Lee and Harris, 2010; Mind, 2022; National Health Service [NHS], 2022; Priory, 2025; Rehab 4 Addiction, 2025), Spain (Gabarda-Blasco et al., 2024; Ortega, 2004; Torres-Soto et al., 2021), Canada (Oke and Kanigsberg, 1991; St-Amour et al., 2024), Sweden (Enoksson et al., 2021; Helleman et al., 2018), Germany (Sayk et al., 2025), Iran (Soleimani et al., 2024), Italy (De Girolamo et al., 2024), Netherlands (Helleman et al., 2014), Portugal (Lima, 2008) and Switzerland (Bromundt et al., 2013).

**Table 1.** Record characteristics.

Author (year)	Country	Record category	Record type	Record aim	Sample	Diagnosis	Sex, age
Borderline in the ACT (2025)	AUS	Educational material	Web page	To describe tips for coping with difficult emotions day by day and improving long-term health and well-being.	N/A	BPD	N/A
Brogan et al. (2020)	US	Research	Case report	To illustrate a case of clinical remission following medication tapering and participation in the 44-day Vital Mind Reset (VMR) online programme.	*N* = 1	Mixed, BPD traits	Female (100%), 40 years
Bromundt et al. (2013)	CH	Research	Pre- and post-test with a control group	To investigate circadian rhythms, sleep and well-being in women with and without light therapy.	BPD *N* = 14; Ctrl. *N* = 10	BPD	BPD female (100%), Mean 30.1 (SD 6) Range 23-41
Clark et al. (2006)	UK	Research	Mixed methods	To describe the planning, implementation and evaluation of a women’s community living skills course.	*N* = 5	BPD	Female (100%), Adult
De Girolamo et al. (2024)	IT	Research	RCT protocol	To test the efficacy of a structured physical activity programme as an additional treatment for BPD.	[*N* = 64]	BPD	(Female [100%], Range 18-40]
Department of Health and Aged Care (2024)	AUS	Programme summary	Web page	To commission psychosocial support services for people who are not accessing similar supports through other programmes.	N/A	Mixed, incl. BPD	N/A
Emotions Matter (2020)	US	Educational material	Leaflet	To share how people with lived experience of BPD are managing daily life during the COVID-19 pandemic.	N/A	BPD	N/A
Enoksson et al. (2021)	SE	Research	Qualitative	To explore experiences of how brief admission influences daily life functioning among people diagnosed with BPD and SH behaviour.	*N* = 16	BPD, SH	Female (87.5%), Mean 32.5 Range 21-44
Gabarda-Blasco et al. (2024)	ES	Research	Pragmatic controlled longitudinal trial	To evaluate Adventure Therapy (AT) with TAU based on CBT.	AT *N* = 26; TAU *N* = 10	BPD	AT female (65.4%), Mean 39.5 Range 31-45; TAU female (60%), Mean 39 Range 29-51
Healthtalk Australia (2025)	AUS	Educational material	Web page	To explore how people negotiate complex experiences of living with a diagnosis of BPD.	N/A	BPD	N/A
Helleman et al. (2014)	NL	Research	Qualitative	To describe the lived experiences of patients diagnosed with BPD who use the brief admission intervention.	*N* = 17	BPD	Female (94.1%), Mean 42.1 Range 28–59
Helleman et al. (2018)	SE	Research	Qualitative	To investigate experiences, strengths and limitations of brief admission and gain knowledge to facilitate its implementation at other treatment centres.	*N* = 8	BPD	Female (100%), Mean 31 Range 23–48
John Hopkins Medicine (2025)	US	Educational material	Web page	To provide patient education about BPD.	N/A	BPD	N/A
Lamont et al. (2017)	UK	Research	Qualitative	To explore the factors underlying the success of four collaborative mental health soccer projects and the role these play in service delivery and personal recovery.	Total *N* = 18; BPD *N* = 2	Mixed, incl. BPD	Total female (22%), Mean 36.7 (SD 10.6) Range 21-64
Lee and Harris (2010)	UK	Research	Mixed methods	To describe the implementation of an OT assessment and treatment pathway.	*N* = 23	BPD	Female (100%), Adult
Lee et al. (2014)	AUS	Research	Mixed methods	To explore the experience of care and outcomes for people entering a bed-based step-up/step-down PARC.	Total *N* = 118; BPD *N* = 13	Mixed, incl. BPD	Total female (48.3%), Mean 40.7 (SD 12.1)
Life Adjustment Team (2025)	US	Programme summary	Web page	To summarise tailored treatment approaches for people diagnosed with PDs.	N/A	Mixed, incl. BPD	N/A
Lima (2008)	PT	Research	Case report	To describe an OT intervention.	*N* = 1	BPD, depression	Female (100%), 45 years
McAlpine (1991)	US	Research	Pre- and post-test with comparative groups	To determine whether a community-based (CB) outreach model is more effective than day treatment (DT) for community adjustment.	Total post-CB *N* = 50, PD *N* = 5; Total post-DT *N* = 30, PD = 3	Mixed, incl. PD	Post-CB PD female (100%), Range 18-40 +; Post-DT PD female (67%), Range 18-40 +
McLean Hospital (2024)	US	Programme summary	Web page	To help patients and their families understand the structure and treatment of the Gunderson Outpatient Programme.	N/A	BPD	N/A
Mehl (1997)	US	Research	Mixed methods	To furnish guidelines for incorporating art therapy and DBT in a group setting, as well as therapeutic techniques that would assist in daily functioning.	*N* = 11	BPD	Female (100%), Adult
Mind (2022)	UK	Educational material	Web page	To explain BPD/ EUPD, including what it feels like, causes, treatment, support and self-care, as well as tips for friends and family.	N/A	BPD/EUPD	N/A
NAMI (2017)	US	Educational material	Web page	To describe first steps to take in managing BPD for a person recently diagnosed.	N/A	BPD	N/A
NDIS (2025)	AUS	Programme summary	Web page	To recommend psychosocial support for NDIS participants.	*N* = 1	BPD	Male (100%), Adult
Nelson and Condrin (1987)	US	Research	Case report	To illustrate the Vocational Readiness and Independent Living skills (VRIL) programme’s effectiveness in the development and maintenance of vocational readiness and ILS.	*N* = 1	Schizophrenia, BPD	Female (100%), 18 years
NHS (2022)	UK	Programme summary	Web page	To provide information about BPD treatment options.	N/A	EUPD	N/A
Oke and Kanigsberg (1991)	CAN	Research	Case report	To describe MPD, its clinical manifestations, and OT assessment and intervention.	*N* = 1	Mixed, incl. BPD	Female (100%), 25 years
Ortega (2004)	ES	Research	Case report	To describe assessment and treatment based on a functional behaviour analysis.	*N* = 1	BPD	Male (100%), 29 years
Orygen (2024)	AUS	Programme summary	Web page	To describe a prevention and early intervention programme for young people diagnosed with BPD.	N/A	BPD	N/A
Pacific Teen Treatment (2024)	US	Programme summary	Web page	To summarise tailored treatment approaches for adolescents diagnosed with BPD.	N/A	BPD	N/A
Priory (2025)	UK	Programme summary	Web page	To summarise treatment services for BPD/EUPD.	N/A	EUPD	N/A
Project Air (2018)	AUS	Educational material	Leaflet	To highlight the importance of self-care and to provide general tips for a healthy lifestyle.	N/A	PD	N/A
Project Air (2020)	AUS	Educational material	Leaflet	To share information created by people with lived experience of a PD diagnosis for what has supported them in navigating challenging times.	N/A	PD	N/A
RANZCP (2017)	AUS & NZ	Educational material	Leaflet	To provide information and advice about BPD based on scientific evidence.	N/A	BPD	N/A
Rehab 4 Addiction (2025)	UK	Educational material	Web page	To provide education about BPD/EUPD treatment.	N/A	BPD	N/A
Sane (2022)	AUS	Educational material	Web page	To provide information about BPD.	N/A	BPD	N/A
Sayk et al. (2025)	DE	Research	Non-randomised controlled (pilot)	To investigate imagery rehearsal therapy (IRT) in people diagnosed with BPD for nightmare symptoms and sleep compared with TAU based on DBT.	IRT *N* = 22; TAU *N* = 22	BPD	IRT–female (100%), Mean 29.91 (SD 9.61); TAU–female (100%), Mean 34.52 (SD 13.13)
Scanlan et al. (2017)	AUS	Research	Mixed methods	To evaluate a peer-delivered hospital transition and post-discharge support programme.	Total *N* = 38; BPD *N* = 3	Mixed; BPD	Total female (47.4%), Mean 45.6 (SD 11.2)
Sekharan et al. (2021)	AUS	Research	Retrospective cohort	To describe the socio-demographic, clinical characteristics and clinical and recovery outcomes of patients admitted to a PARC service.	BPD *N* = 61; BPD traits *N* = 6	BPD; BPD traits	Female (85.1%), Mean 37.27 (SD 10.9)
Soleimani et al. (2024)	IR	Research	Pre- and post-test with control group	To examine coping skills training on schemas, health-promoting behaviours and mental well-being.	N/A	BPD	Female (65%)
St-Amour et al. (2024)	CAN	Research	Mixed methods	To examine the effect of a structured physical exercise programme on emotion regulation.	*N* = 7	BPD	Female (100%), Mean 32.43 (SD 7.79) Range 23-45
Tomasi et al. (2019)	US	Research	Pre- and post-test with a single group	To promote exercise, fitness and physical health in inpatient psychiatry patients.	*N* = 100	Mixed, incl. BPD	N/A
Torres-Soto et al. (2021)	ES	Research	Longitudinal, pre- and post-test	To establish whether the Day Hospital treatment programme is effective.	*N* = 100; BPD *N* = 60	Mixed PD; BPD	Total female (73%), Range 18-54
verywell mind (2023)	US	Educational material	Web page	To outline coping skills that can help to reduce emotion dysregulation and other symptoms of BPD.	N/A	BPD	N/A

AUS = Australia; BPD = borderline personality disorder; AT = Adventure Therapy; CAN = Canada; CB = community-based; CBT = cognitive behavioural therapy; CH = Switzerland; Ctrl. = Control; DBT = dialectical behaviour therapy; DE = Germany; DT = day treatment; EUPD = emotionally unstable personality disorder; ES = Spain; ILS = independent living skills; MPD = multiple personality disorder; *N*/A = not available; NAMI = National Alliance on Mental Illness; NDIS = National Disability Insurance Scheme; NHS = National Health Service; NL = Netherlands; NZ = New Zealand; OT = Occupational Therapy; PARC = Prevention and Recovery Centre; PD = personality disorder; PT = Portugal; RANZCP = Royal Australian & New Zealand College of Psychiatrists; RCT = randomised control trial; SD = standard deviation; SE = Sweden; SH = self-harming; TAU = treatment as usual; UK = United Kingdom; US = United States.

There were three types of records: research, educational materials and programme summaries. The research records were controlled studies (Bromundt et al., 2013; De Girolamo et al., 2024; Gabarda-Blasco et al., 2024; McAlpine, 1991; Sayk et al., 2025; Sekharan et al., 2021; Soleimani et al., 2024), mixed methods (Clark et al., 2006; Lee et al., 2014; Lee and Harris, 2010; Mehl, 1997; Scanlan et al., 2017; St-Amour et al., 2024), descriptive studies (Brogan et al., 2020; Lima, 2008; Nelson and Condrin, 1987; Oke and Kanigsberg, 1991; Ortega, 2004), qualitative studies (Enoksson et al., 2021; Helleman et al., 2014, 2018; Lamont et al., 2017) and uncontrolled studies (Tomasi et al., 2019; Torres-Soto et al., 2021). The educational materials were web pages (Borderline in the ACT, 2025; Healthtalk Australia, 2025; John Hopkins Medicine, 2025; Mind, 2022; NAMI, 2017; Rehab 4 Addiction, 2025; Sane, 2022; verywell mind, 2023) and leaflets (Emotions Matter, 2020; Project Air, 2018, 2020; RANZCP, 2017). The programme summaries were web pages (Department of Health and Aged Care, 2024; Life Adjustment Team, 2025; McLean Hospital, 2024; NDIS, 2025; Orygen, 2024; Pacific Teen Treatment, 2024; Priory, 2025).

The BPD diagnosis sample size (*n* = 340) was derived from records describing people with the diagnosis only (Brogan et al., 2020; Clark et al., 2006; Enoksson et al., 2021; Gabarda-Blasco et al., 2024; Helleman et al., 2014, 2018; Lee and Harris, 2010; Lima, 2008; Mehl, 1997; NDIS, 2025; Nelson and Condrin, 1987; Oke and Kanigsberg, 1991; Ortega, 2004; Sayk et al., 2025; Sekharan et al., 2021; St-Amour et al., 2024) or records that reported the cohort separately (Bromundt et al., 2013; Lamont et al., 2017; Lee et al., 2014; McAlpine, 1991; Scanlan et al., 2017; Torres-Soto et al., 2021). This BPD diagnosis sample excludes the educational materials (outlined above), one record that did not report the cohort separately (Tomasi et al., 2019) and one record outlining a study protocol (De Girolamo et al., 2024). The sex distribution of the BPD diagnosis sample was 84% female and 16% male, calculated either from direct reporting (Brogan et al., 2020; Bromundt et al., 2013; Clark et al., 2006; Enoksson et al., 2021; Gabarda-Blasco et al., 2024; Helleman et al., 2014, 2018; Lee and Harris, 2010; Lima, 2008; McAlpine, 1991; Mehl, 1997; NDIS, 2025; Nelson and Condrin, 1987; Oke and Kanigsberg, 1991; Ortega, 2004; Sayk et al., 2025; Sekharan et al., 2021; St-Amour et al., 2024) or conservatively estimated using whole-sample proportions applied to the BPD diagnosis subset (Lamont et al., 2017; Lee et al., 2014; Scanlan et al., 2017; Torres-Soto et al., 2021). The age range of the participants in the directly reported BPD diagnosis sample was 18 to 59 years.

### Programme aims, setting and structure

#### Programme aims

Thirty-two records described 34 facilitator-led programmes that addressed daily living functioning, as outlined in Table 2. The educational materials were removed from this table because they lacked a programme structure and facilitator. Programmes addressed daily living functioning through independent living skills development (Clark et al., 2006; Department of Health and Aged Care, 2024; Life Adjustment Team, 2025; McAlpine, 1991; NDIS, 2025; Nelson and Condrin, 1987), exercise and nutrition (De Girolamo et al., 2024; Lamont et al., 2017; St-Amour et al., 2024; Tomasi et al., 2019), residential facilities (Lee et al., 2014; NHS, 2022; Pacific Teen Treatment, 2024; Priory, 2025; Sekharan et al., 2021), brief admission to hospital (Enoksson et al., 2021; Helleman et al., 2014, 2018), outpatient treatment (McLean Hospital, 2024; Priory, 2025; Torres-Soto et al., 2021), occupational therapy (Lee and Harris, 2010; Lima, 2008; Oke and Kanigsberg, 1991), adventure-based therapy (Gabarda-Blasco et al., 2024), art therapy (Mehl, 1997), coping skills (Soleimani et al., 2024), early intervention (Orygen, 2024), functional behaviour analysis (Ortega, 2004), imagery rehearsal therapy (Sayk et al., 2025), light therapy (Bromundt et al., 2013), promotion of lifestyle changes (Brogan et al., 2020) and transition from hospital to home (Scanlan et al., 2017).

**Table 2.** Aims and structure of programmes.

Author (year)	Programme	Programme aim	Approach	Mode	Setting	Frequency	Duration	Facilitator(s)
Brogan et al. (2020)	Vital Mind Reset (VMR)	Focus on lifestyle changes, diet, meditation and supplements.	Mind-body medicine	Indv	Online	ND	44 d	MD, Researcher, Med. Student & NatD
Bromundt et al. (2013)	Light Therapy	Consolidate circadian sleep-wake cycles to improve mood, daytime alertness and sleep quality.	Chronobiology/ sleep	Indv	HB (winter)	Daily (mornings)	3 w	Researcher
Clark et al. (2006)	Community Living Skills Course	Empower and build skills for women living in a residential facility to transition safely into the community.	Recovery & empowerment	Grp	CB	Weekly, 2 h sess.	10 sess.	OT, PSY Assist., OT Tech. Instructor & Student OT
Department of Health and Aged Care (2024)	Commonwealth Psychosocial Support Programme (CPSP)	Work in partnership with consumers (alongside families and carers) to achieve recovery goals.	Recovery	Grp & Indv	CB	ND	ND	Primary health care orgs.
Di Girolamo et al. (2024)	Structured physical activity intervention	Test the efficacy of physical activity as an additional treatment.	Physical activity & mind-body medicine	Grp	SC	1 h sess., 3x p/w	12 w (36 sess.)	Sports Physician & Personal Trainer
Di Girolamo et al. (2024)	Psychoeducation programme	Promote a healthy diet, lifestyle choices and physical activity while highlighting the risks of sedentary behaviour.	Psychoeducation & mind-body medicine	Grp	RSC	1 h sess., weekly	8 sess.	Nutritionist, PSY
Enoksson et al. (2021) & Helleman et al. (2014, 2018)^a^	Brief admission (to hospital)	Increase responsibility, coping and quality of life.	Crisis mgmt./ autonomy	Indv	IPU	ND	1-3 nights	Nurse
Gabarda-Blasco et al. (2024)	Adventure Therapy (AT)	Use adventure to bring about changes in different areas of life.	Exper. learning/ ABT	Grp	OD	2 h sess, weekly	14 w	Therapist (ND)
Lamont et al. (2017)	Football & walking football (soccer) groups	Improve sense of self via collaborative sports.	Recovery & physical activity	Grp	CB & IPU	Weekly	Football (soccer) game	Service User, MHN, Physio., SupW & Vol.
Lee et al. (2014) & Sekharan et al.(2021)^b^	Admission to a Prevention & Recovery Centre (PARC)	Prepare for a return to community living or to prevent psychiatric hospital admission.	Recovery & stepped care	Grp & Indv	RF	ND	Up to 28 d	ND
Lima (2008)	Occupational Therapy	Integrate into the community by focusing on environment, self-care and leisure.	Rehab.	Indv	HB	Fortnightly	1 y	OT
McAlpine (1991)	Sharing Life in the Community Programme (SLIC)	Increase self-reliance and reduce dependence on hospitals and other institutions.	Rehab.	Indv	CB	1 h sess., 1-4 x p/w & 24 h crisis serv.	6-12 mo.	MD, PSY, SW, OT, Nurse & SupW
McLean Hospital (2024)	Gunderson Outpatient Programme	Emphasise the psychological, relationship and school/work issues that arise as patients return to their lives in the community.	Rehab. & PT	Grp & Indv	OPD	Weekly: CM (.5 h), Indv PT (1-2 sess.), Grp PT (8-10 to 1-2 sess.) & FT	ND	CM, PT, FT & programme/clinical staff
Mehl (1997)	Art Therapy	Provide clients with new coping skills and enable a sense of proficiency in managing everyday life events.	Art & PT	Grp	OPD	Weekly, 2 h	12 sess. (often repeated)	Art Therapist
NDIS (2025)	Psychosocial supports	Support to achieve personal recovery, increase independence and inclusion in the community.	Recovery & rehab.	Grp & Indv	CB	ND	ND	PsyRecNav & serv. provider
Nelson and Condrin (1987)	Vocational Readiness & Independent Living skills (VRIL)	Meet the vocational and independent living skills training needs of adolescents with psychiatric impairment.	Rehab. & systems theory	Grp & Indv	School & IPU	5 d p/w	ND	Child Devp., OT, MD, Nurse, RD, Paed., RT, SW, VR & SE
NHS (2022)	Therapeutic Communities (TCs)	Improve social skills and self-confidence.	Recovery & rehab.	Grp & Indv	RF	1-4 d p/w	ND	Staff & residents
Oke and Kanigsberg (1991)	Occupational Therapy	Support an individual’s ability to function independently in the areas of self-care, productivity and leisure.	Rehab. & devp. frame of ref.	Indv	ND	ND	ND	OT
Ortega (2004)	Functional behaviour analysis (FBA)	To modify behavioural variables (prohibit coffee/caffeine intake).	Behav. analysis & mod.	Indv	OPD & RF	Weekly, 1 h sess.	ND	PT
Orygen (2024)	Helping Young People Early (HYPE) [Grp Programme]	Provide early intervention treatment for young people diagnosed with BPD.	Early intervention	Grp	CB	Weekly, 1-2 h	ND	ND
Pacific Teen Treatment (2024)	[Finding help for borderline personality disorder]	Provide a tailored treatment plan unique to each young person’s needs.	Rehab. & PT	Grp & Indv	RF	Daily, Grp & Indv PT	30–60 d	ND
Priory (2025)	[Inpatient treatment] Therapeutic Communities (TCs)	Have respite from the usual routine and form healthier day-to-day habits.	Rehab & PT	Grp & Indv	RF	24 h support	ND	Therapist (ND)
Priory (2025)	Outpatient and day care	Offer flexible, ongoing support and transition back to the usual routine.	PT & transitional care	Grp & Indv	OPD	1 h to day-long	ND	Therapist (ND) & consultant (ND)
Sayk et al. (2025)	Imagery Rehearsal Therapy (IRT)	Treat nightmares to improve sleep.	PT & chronobiology/ sleep	Grp	IPU	Weekly	8 sess.	PSY
Scanlan et al. (2017)	Hospital to Home (H2 H)	Link prior to discharge from hospital and provide post-discharge support.	Transitional care	Indv	CB	ND	6-8 w	Peer Worker
Soleimani et al. (2024)	Coping Skills Training Programme	Reduce maladaptive schemas and increase health-promoting lifestyle behaviours.	PT	Grp	OPD	1.5 h, 2x p/w	12 sess.	ND
St-Amour et al. (2024)	Supervised physical exercise	Examine the effect of structured physical exercise on emotions and sleep quality.	Physical activity	Indv	HB & online	1 h, 3x p/w	4 w (12 sess.)	Research assistant (ND), MD resident & PSY student
Tomasi et al. (2019)	Exercise & nutrition groups	Offer exercise groups & nutrition education sessions.	Mind-body medicine	Grp	IPU	4x p/w, 1 h ex. & 1 h nut.	ND	PT, GT, RN & MH Tech.
Torres-Soto et al. (2021)	Day Hospital treatment programme	Achieve containment of the most serious symptoms and attain sufficient symptomatic and psychosocial stabilisation.	Symptom mgmt. & Rehab.	Grp & Indv	OPD	ND	ND	MD, PSY, RN & SW

ABT = adventure-based therapy; assist. = assistant; AT = Adventure Therapy; behav. = behavioural; BPD = borderline personality disorder; CB = community-based; CM = case management/manager; d = day(s); Dept. = department; devp. = development(al); exper. = experiential; FT = family therapy/therapist; Grp = group; GT = group therapist; h = hour(s); HB = home-based; Indv = individual; IPU = inpatient psychiatric unit; m = minute(s); MD = psychiatry/psychiatrist; med. = medical; mgmt. = management; MH = mental health; MHN = mental health nurse; mo. = month(s); mod. = modification; ND = not described; NatD = naturopathic physician; OD = outdoors; OPD = outpatient treatment department/program; OT = occupational therapy/therapist; orgs. = organisations; p/w = per week; Paed. = paediatrics/paediatrician; physio. = physiotherapist; PSY = psychology/psychologist; PsyRecNav = psychosocial recovery navigator; PT = psychotherapy/psychotherapist; Rehab. = rehabilitation; RD = dietetics/dietician; ref. = reference; RF = residential facility; RN = registered nurse; RSC = research site centre; RT = recreation therapist; SC = sports centre; SE = special education; sess. = session(s); serv. = service; SW = social work/worker; SupW = support worker/aide; tech. = technical/technician; vol. = volunteer; VR = vocational rehabilitator; w = week(s); x = times; y = year(s).

a Three studies on the same programme.

b Two studies on the same programme. Programme setting.

Programmes were delivered in the community (Clark et al., 2006; Department of Health and Aged Care, 2024; Lamont et al., 2017; McAlpine, 1991; NDIS, 2025; Orygen, 2024; Scanlan et al., 2017), inpatient units (Enoksson et al., 2021; Helleman et al., 2014, 2018; Lamont et al., 2017; Lee and Harris, 2010; Sayk et al., 2025; Tomasi et al., 2019), outpatient treatment centres (McLean Hospital, 2024; Mehl, 1997; Ortega, 2004; Priory, 2025; Soleimani et al., 2024; Torres-Soto et al., 2021), residential facilities (Lee et al., 2014; NHS, 2022; Pacific Teen Treatment, 2024; Priory, 2025; Sekharan et al., 2021), at home (Bromundt et al., 2013; Life Adjustment Team, 2025; Lima, 2008; St-Amour et al., 2024), online (Brogan et al., 2020; St-Amour et al., 2024), research facilities (De Girolamo et al., 2024), special schools (Nelson and Condrin, 1987), sports centres (De Girolamo et al., 2024) and outdoors (Gabarda-Blasco et al., 2024). One record did not describe the programme setting (Oke and Kanigsberg, 1991).

#### Programme structure

Programmes were implemented individually (Brogan et al., 2020; Bromundt et al., 2013; Enoksson et al., 2021; Helleman et al., 2014, 2018; Life Adjustment Team, 2025; Lima, 2008; McAlpine, 1991; Oke and Kanigsberg, 1991; Ortega, 2004; Scanlan et al., 2017; St-Amour et al., 2024) and in groups (Clark et al., 2006; De Girolamo et al., 2024; Gabarda-Blasco et al., 2024; Lamont et al., 2017; Lee and Harris, 2010; Mehl, 1997; Orygen, 2024; Sayk et al., 2025; Soleimani et al., 2024; Tomasi et al., 2019). Programmes also combined individual and group components (Department of Health and Aged Care, 2024; Lee et al., 2014; McLean Hospital, 2024; NDIS, 2025; Nelson and Condrin, 1987; NHS, 2022; Pacific Teen Treatment, 2024; Priory, 2025; Sekharan et al., 2021; Torres-Soto et al., 2021). Programme facilitators were diverse, including consumers (service users), nurses, nutritionists, occupational therapists, peer workers, personal trainers, physiotherapists, psychiatrists, psychologists, researchers, social workers, students and support workers. Programme duration varied greatly, ranging from eight sessions to 12 months.

### Approaches underpinning programmes

Programmes typically utilised multiple approaches, as shown in Table 2. The most frequently used approaches were rehabilitation (Lee and Harris, 2010; Life Adjustment Team, 2025; Lima, 2008; McAlpine, 1991; McLean Hospital, 2024; NDIS, 2025; Nelson and Condrin, 1987; NHS, 2022; Oke and Kanigsberg, 1991; Pacific Teen Treatment, 2024; Priory, 2025; Torres-Soto et al., 2021) and recovery (Clark et al., 2006; Department of Health and Aged Care, 2024; Lamont et al., 2017; Lee et al., 2014; NDIS, 2025; NHS, 2022; Sekharan et al., 2021), followed by psychotherapy (McLean Hospital, 2024; Mehl, 1997; Pacific Teen Treatment, 2024; Priory, 2025; Sayk et al., 2025; Soleimani et al., 2024), physical activity (De Girolamo et al., 2024; Gabarda-Blasco et al., 2024; Lamont et al., 2017; St-Amour et al., 2024), mind-body medicine (Brogan et al., 2020; De Girolamo et al., 2024; Tomasi et al., 2019), crisis management/autonomy (Enoksson et al., 2021; Helleman et al., 2014, 2018), stepped care (Lee et al., 2014; Sekharan et al., 2021), transitional care (Priory, 2025; Scanlan et al., 2017), chronobiology/sleep (Bromundt et al., 2013; Sayk et al., 2025), adventure-based therapy (Gabarda-Blasco et al., 2024), art therapy (Mehl, 1997), behaviour analysis/modification (Ortega, 2004), developmental frame of reference (Oke and Kanigsberg, 1991), early intervention (Orygen, 2024), empowerment (Clark et al., 2006), experiential learning (Gabarda-Blasco et al., 2024), psychoeducation (De Girolamo et al., 2024), symptom management (Torres-Soto et al., 2021) and systems theory (Nelson and Condrin, 1987).

### Domains of daily living functioning addressed in records

Table 3 summarises the domains of daily living functioning addressed across all 44 records, including the self-directed educational materials. The most frequently addressed domain was ‘health’ (*n* = 189), followed by ‘relational’ (*n* = 84), ‘responsibility’ (*n* = 67), ‘personal’ (*n* = 61), ‘leisure’ (*n* = 53), ‘routine’ (*n* = 42) and ‘household’ (*n* = 30).

**Table 3.** Domains of daily living functioning addressed in records.

Record	Health												Relational				Responsibility						Personal						Leisure				Routine			Household		
	Physical activity	Psychotherapy engagement	Healthcare utilisation	Crisis management	Nutrition	Medication adherence	Sleep hygiene	Psychoeducation	Self-harm behaviours	Personal hygiene	Stress management	Substance use	Social skills	Social connection	Peer support	Family relationships	Personal autonomy	Vocational engagement	Financial management	Community participation	Digital technology	Pet care	Emotional regulation	Adaptive strategies	Identity development	Self-efficacy	Motivation	Sense of security	Recreational activities	Creative expression	Mindfulness practice	Rest	Routine management	Task organisation	Goal setting	Domestic maintenance	Cooking and shopping	Housing stability
1	x	x	x	x	x	x				x		x	x	x	x								x	x					x		x	x						
2			x		x	x						x		x		x	x	x			x		x		x						x							
3							x																x										x					
4				x				x					x	x	x		x		x		x					x							x		x		x	
5	x											x	x	x		x		x	x							x							x			x	x	x
6	x				x			x																														
7	x			x	x		x	x	x	x	x			x	x	x	x	x			x			x					x	x	x	x	x	x	x	x		
8	x		x	x	x		x		x		x			x		x	x	x				x	x	x		x						x	x	x				
9	x												x		x		x			x			x		x													
10	x		x			x		x	x	x	x		x	x	x	x		x		x		x	x		x				x	x	x		x	x		x		
11	x	x	x	x	x	x	x		x	x			x	x	x		x						x			x		x	x			x	x	x				
12	x		x	x	x	x	x		x				x	x	x		x						x	x				x		x		x	x	x				
13	x	x	x	x	x	x	x	x	x		x	x				x																						
14	x												x	x	x		x	x											x				x					
15		x							x	x			x	x				x		x			x	x	x		x		x	x			x	x		x	x	
16		x				x	x	x			x		x	x			x	x											x	x	x						x	x
17		x		x		x				x	x		x			x		x		x			x	x	x								x		x	x		
18						x				x			x			x		x	x		x		x						x			x	x	x	x	x	x	
19			x	x		x				x			x	x				x	x	x			x		x				x							x	x	x
20	x	x		x	x		x						x				x	x	x	x			x	x							x		x					
21				x			x											x												x			x	x				
22	x	x		x	x	x	x	x	x	x	x	x	x	x	x				x	x	x		x	x					x	x	x	x						
23	x	x	x		x					x	x	x		x	x									x														
24			x					x					x		x	x		x			x																	x
25			x	x						x			x					x	x	x									x							x	x	
26		x							x			x	x		x											x			x							x	x	
27			x		x								x	x			x	x					x		x	x			x	x				x			x	
28		x										x	x	x																								
29	x		x								x	x	x	x	x		x	x					x			x			x	x				x				
30	x	x			x	x	x																x								x	x	x					
31	x	x				x		x	x				x	x	x								x		x	x			x	x	x		x		x	x		
32	x				x		x					x																						x				
33	x	x	x	x				x		x			x	x	x	x	x				x			x	x				x	x	x	x	x		x		x	
34		x	x	x				x					x	x		x			x				x	x														
35	x	x	x	x		x							x	x	x								x							x	x						x	
36	x	x	x	x	x		x	x	x			x	x		x	x	x						x	x						x	x		x		x			
37							x										x						x															
38			x		x									x	x		x		x	x	x															x	x	
39		x	x	x		x			x			x	x				x	x	x						x				x				x		x			x
40	x	x									x		x	x		x	x			x			x	x		x	x		x		x				x		x	
41	x						x				x												x						x					x				
42	x		x		x														x																		x	
43		x	x	x		x		x	x	x			x	x												x								x				
44	x	x		x									x	x										x	x					x	x			x		x		
Ttl	24	20	20	19	16	15	15	13	13	12	11	11	29	25	17	13	18	18	11	10	8	2	23	13	11	10	2	2	18	14	13	8	19	14	9	12	13	5
	189												84				67						61						53				42			30		

Domains of daily living functioning adapted from Desrosiers et al. (2017).

1 = Borderline in the ACT, 2025, 2 = Brogan et al., 2020, 3 = Bromundt et al., 2013, 4 = Clark et al., 2006, 5 = Department of Health and Aged Care, 2024, 6 = De Girolamo et al., 2024, 7 = Emotions Matter, 2020, 8 = Enoksson et al., 2021, 9 = Gabarda-Blasco et al., 2024, 10 = Healthtalk Australia, 2025, 11 = Helleman et al., 2014, 12 = Helleman et al., 2018, 13 = John Hopkins Medicine, 2025, 14 = Lamont et al., 2017, 15 = Lee and Harris, 2010, 16 = Lee et al., 2014, 17 = Life Adjustment Team, 2025, 18 = Lima, 2008, 19 = McAlpine, 1991, 20 = McLean Hospital, 2024, 21 = Mehl, 1997, 22 = Mind, 2022, 23 = NAMI, 2017, 24 = NDIS, 2025, 25 = Nelson and Condrin, 1987, 26 = NHS, 2022, 27 = Oke and Kanigsberg, 1991, 28 = Ortega, 2004, 29 = Orygen, 2024, 30 = Pacific Teen Treatment, 2024, 31 = Priory, 2025, 32 = Project Air, 2018, 33 = Project Air, 2020, 34 = RANZCP, 2017, 35 = Rehab 4 Addiction, 2025, 36 = Sane, 2022, 37 = Sayk et al., 2025, 38 = Scanlan et al., 2017, 39 = Sekharan et al., 2021, 40 = Soleimani et al., 2024, 41 = St-Amour et al., 2024, 42 = Tomasi et al., 2019, 43 = Torres-Soto et al., 2021, 44 = verywell mind, 2023.

### Measures used to describe changes in functioning

Eighty-nine measures were used to describe changes in functioning across the included quantitative studies (Brogan et al., 2020; Bromundt et al., 2013; Clark et al., 2006; De Girolamo et al., 2024; Gabarda-Blasco et al., 2024; Lee et al., 2014; Lee and Harris, 2010; Lima, 2008; McAlpine, 1991; Mehl, 1997; Nelson and Condrin, 1987; Ortega, 2004; Sayk et al., 2025; Scanlan et al., 2017; Sekharan et al., 2021; Soleimani et al., 2024; St-Amour et al., 2024; Tomasi et al., 2019; Torres-Soto et al., 2021). Sixty-four of these were valid and reliable, such as the Borderline Evaluation of Severity Over Time (BEST) (Pfohl et al., 2009), Kohlman Evaluation of Living Skills (KELS) (Burnett et al., 2009) and Recovery Assessment Scale – Domains and Stages (RAS-DS) (Hancock et al., 2015). The Health of the Nation Outcome Scale (HoNOS) appeared in two separate studies of the Prevention & Recovery Centre (PARC) in Victoria, Australia (Lee et al., 2014; Sekharan et al., 2021), despite its lack of reliability and validity (Brooks, 2000). An overview of these measures is provided in Supplementary File 1. The wide array of measures demonstrates the lack of standardised assessment of daily living functioning.

## Discussion

This scoping review synthesised literature describing programmes that address the daily living functioning of people diagnosed with BPD. Persistent functional impairment despite symptomatic remission represents a critical gap in current treatment (Álvarez-Tomás et al., 2019; Choi-Kain et al., 2020). The programmes found in this review were bespoke and service-specific, with considerable variability in aims, content and delivery. This ad hoc approach to addressing daily living functioning is in stark contrast to the theoretically informed and manualised psychotherapies (Choi-Kain et al., 2017; Storebø et al., 2020). Such variability in rehabilitation for daily living functioning indicates the need for a more systematic and evidence-based approach.

The high frequencies of the ‘relational’ and ‘responsibilities’ domains of functioning found in this review reflect the well-documented challenges that people diagnosed with BPD face in employment and relationships (Grenyer et al., 2022; Miller et al., 2018; Zanarini et al., 2010b). The other frequently occurring domains seemed to highlight burgeoning areas of interest in research, including ‘sleep hygiene’ (King et al., 2024), ‘healthcare utilisation’ (Botter et al., 2021; Glass et al., 2024), ‘physical activity’ (Petersen et al., 2025), ‘nutrition’ (St-Amour et al., 2023), ‘crisis management’ (Warrender et al., 2020), ‘personal hygiene’ (Potvin et al., 2019), ‘routine management’ (Birken and Harper, 2017; Falklöf and Haglund, 2010) and ‘recreational activities’ (leisure) (Borovica et al., 2024). Importantly, greater focus on these ‘other’ domains of functioning could support people diagnosed with BPD in obtaining and maintaining work (Kernot et al., 2023; Larivière et al., 2025).

Measures used to describe changes in functioning were strikingly heterogeneous, with 89 measures identified, 25 (28%) of which were bespoke or lacked reliability and validity. This large number of measures reflects the disparate nature of the evidence base informing rehabilitation programmes for people diagnosed with BPD. The diversity of measures also reflects the broader challenge of assessing functional impairment in mental health (Üstün and Kennedy, 2009). The Global Assessment of Functioning (GAF) (Endicott et al., 1976) was not used to describe changes in functioning in any of the programmes, confirming its lack of clinical utility (Gold, 2014). Notably, while there is a measure of daily living functioning specifically designed for people diagnosed with BPD, it is currently only available in French (Desrosiers et al., 2017).

### Recommendations for practice

Given persistent functional impairment, rehabilitation for daily living functioning should be offered alongside or re-centred within evidence-based psychotherapy for people diagnosed with BPD. Rehabilitation could involve greater emphasis on the domains of daily living functioning outlined in Table 3, including health (sleep hygiene, physical activity and nutrition), household management (cooking and shopping), routine management (task organisation), responsibility (financial management and community participation) and leisure (recreational activities and creative expression). This approach requires shifting focus to consider all domains of daily living functioning, rather than relying solely on relational and vocational domains as indicators of functional recovery. Practitioners can draw from the available rehabilitation programmes and measures found in this review to structure and evaluate service delivery. However, practitioners will need to rely on clinical judgement when choosing between options, as a robust and comparable research evidence base has not yet been established.

### Recommendations for future research

Research is needed to strengthen the evidence base for rehabilitation programmes addressing the daily living functioning of people diagnosed with BPD. This involves developing and rigorously evaluating comprehensive programmes that address all domains of functioning. A validated English-language assessment tool for evaluating daily living functioning is urgently needed. The development of such a tool and future programmes addressing daily living functioning should be informed by both lived experience and clinical perspectives. Future research should evaluate whether addressing daily living functioning improves both psychotherapy and sustained work outcomes.

### Strengths and limitations

A strength of this review is its comprehensive scope, with no date or language restrictions and incorporating grey literature across multiple countries. However, programmes reporting positive outcomes are more likely to be available, creating publication bias. The search terms were based on a Google Translate version of Desrosiers et al.’s (2017) assessment tool, potentially excluding programmes using different terminology to describe daily living functioning. The predominance of female participants (84%) in the sample may limit the generalisability of findings to males diagnosed with BPD (Larivière et al., 2023).

## Conclusion

Rehabilitation for daily living functioning is critically important for people diagnosed with BPD given persistent functional impairment, yet a comprehensive search of existing programmes found only 44 records to guide service delivery. There were substantial inconsistencies in the aims, content and delivery of these programmes. Daily living functioning requires increased research attention and clinical focus to enable functional recovery. Future rehabilitation programmes must comprehensively address all domains of daily living functioning and be systematically evaluated using reliable and valid measures.

## Supplemental Material

sj-docx-1-anp-10.1177_00048674251393159 – Supplemental material for A scoping review of programmes that address the daily living functioning of people diagnosed with borderline personality disorder Supplemental material, sj-docx-1-anp-10.1177_00048674251393159 for A scoping review of programmes that address the daily living functioning of people diagnosed with borderline personality disorder by Dillon Tepper, Ben Sellar, Sheryl Shipley, Rachel Smith and Carolyn M Murray in Australian & New Zealand Journal of Psychiatry
