# Supplementary material for: A scoping review of programmes that address the daily living functioning of people diagnosed with borderline personality disorder
Source: Aust N Z J Psychiatry. 2025 Nov 29;60(8):747–64. doi: 10.1177/00048674251393159 (PMC13392200; doi:10.1177/00048674251393159)
Supplement: sj-docx-1-anp-10.1177_00048674251393159 – Supplemental material for A scoping review of programmes that address the daily living functioning of people diagnosed with borderline personality disorder [file sj-docx-1-anp-10.1177_00048674251393159.docx]

**Supplementary File 1**

**Title**

A scoping review of programs that address the daily living functioning of people diagnosed with borderline personality disorder

**Search date**

Conducted on the 5^th^ of August 2025.

**Contents**

[Database search 1](#_Toc206875555)

[Medline 1](#_Toc206875556)

[Embase and Emcare 5](#_Toc206875557)

[APA PsycInfo 7](#_Toc206875558)

[CINAHL 11](#_Toc206875559)

[ProQuest and ASSIA 15](#_Toc206875560)

[Scopus 16](#_Toc206875561)

[Grey literature search 17](#_Toc206875562)

[Google and Google Scholar search terms 17](#_Toc206875563)

[BPD organisation websites 17](#_Toc206875564)

[Email template 19](#_Toc206875565)

[Measures used to evaluate functioning 20](#_Toc206875566)

# Database search

## Medline

| Line | Search term | Search type | Scope |
| --- | --- | --- | --- |
| 1 | Borderline Personality Disorder/ | MeSH Heading | A personality disorder marked by a pattern of instability of interpersonal relationships, self-image, and affects, and marked impulsivity beginning by early adulthood and present in a variety of contexts. (DSM-IV). |
| 2 | ((borderline or emotionally unstable) adj1 personality disorder*).ti,ab,kf. | Keyword | N/A |
| 3 | ("F60.3" or "F60.30" or "F60.31").ti,ab,kf. | Keyword | N/A |
| 4 | or/1-3 |  |  |
| 5 | ((self or personal) adj3 (appearance or care or hygiene or manage*)).ti,ab,kf. | Keyword | N/A |
| 6 | Self Care/ | MeSH Heading | Caring for self when ill or positive actions and adopting behaviors to prevent illness. |
| 7 | Laundering/ | MeSH Heading | The process of washing clothes, linens or other fabric. |
| 8 | (laundry or laundering).ti,ab,kf. | Keyword |  |
| 9 | Feeding Behavior/ | MeSH Heading | Behavioral responses or sequences associated with eating including modes of feeding, rhythmic patterns of eating, and time intervals. |
| 10 | ((diet* or eat* or feed* or food) adj3 (behav* or choice* or habit*)).ti,ab,kf. | Keyword | N/A |
| 11 | Physical Fitness/ | MeSH Heading | The ability to carry out daily tasks and perform physical activities in a highly functional state, often as a result of physical conditioning. |
| 12 | Sedentary Behavior/ | MeSH Heading | Behaviors during waking hours that have low energy expenditure and are often performed in a sitting or reclining POSTURE. |
| 13 | (physical adj3 (activit* or condition or exercise* or fitness or wellbeing or well being)).ti,ab,kf. | Keyword |  |
| 14 | (sedentary adj3 (behav* or life*)).ti,ab,kf. | Keyword |  |
| 15 | Sleep Hygiene/ | MeSH Heading | Habits and practices conducive to getting the right amount and quality of sleep, and include responding to environmental factors that may influence one's sleep. |
| 16 | (sleep adj3 (habit* or hygiene or routine* or schedule*)).ti,ab,kf. | Keyword | N/A |
| 17 | Attitude to Health/ | MeSH Heading | Public attitudes toward health, disease, and the medical care system. |
| 18 | Health Behavior/ | MeSH Heading | Combination of HEALTH KNOWLEDGE, ATTITUDES, PRACTICE which underlie actions taken by individuals regarding their health. |
| 19 | Health Literacy/ | MeSH Heading | Degree to which individuals have the capacity to obtain, process, and understand basic health information and services needed to make appropriate health decisions. |
| 20 | Health Status/ | MeSH Heading | The level of health of the individual, group, or population as subjectively assessed by the individual or by more objective measures. |
| 21 | (health adj3 (attitude* or behav* or knowledge* or literacy or maint* or state or status)).ti,ab,kf. | Keyword | N/A |
| 22 | Medication Adherence/ | MeSH Heading | Voluntary cooperation of the patient in taking drugs or medicine as prescribed. This includes timing, dosage, and frequency. |
| 23 | (medication* adj3 (adherence or compliance or manag*)).ti,ab,kf. | Keyword | N/A |
| 24 | Housing/ | MeSH Heading | Living facilities for humans. |
| 25 | ((domestic or home or house* or housing) adj3 (activit* or chore* or clean* or duty or duties or keeping or maint* or manage* or skill* or task*)).ti,ab,kf. | Keyword | N/A |
| 26 | Cooking/ | MeSH Heading | The art or practice of preparing food. It includes the preparation of special foods for diets in various diseases. |
| 27 | Meals/ | MeSH Heading | A portion of the food eaten for the day, usually at regular occasions during the day. |
| 28 | ((cook* or food or grocery or groceries or meal) adj3 (plan* or prep* or shop* or skill*)).ti,ab,kf. | Keyword | N/A |
| 29 | Time Management/ | MeSH Heading | Planning and control of time to improve efficiency and effectiveness. |
| 30 | (time adj3 (manag* or organis* or organiz* or priorit* or structur*)).ti,ab,kf. | Keyword | N/A |
| 31 | Independent Living/ | MeSH Heading | A housing and community arrangement that maximizes independence and self-determination. |
| 32 | independent living.ti,ab,kf. | Keyword | N/A |
| 33 | Fatigue/ | MeSH Heading | The state of weariness following a period of exertion, mental or physical, characterized by a decreased capacity for work and reduced efficiency to respond to stimuli. |
| 34 | (fatigue adj3 manag*).ti,ab,kf. | Keyword | N/A |
| 35 | "Activities of Daily Living"/ | MeSH Heading | The performance of the basic activities of self care, such as dressing, ambulation, or eating. |
| 36 | Habits/ | MeSH Heading | Acquired or learned responses which are regularly manifested. |
| 37 | ((activit* adj3 daily living) or ADL).ti,ab,kf. | Keyword | N/A |
| 38 | ((daily or everyday) adj3 (activit* or function* or habit* or life or living or routine* or schedul* or task*)).ti,ab,kf. | Keyword | N/A |
| 39 | Life Style/ | MeSH Heading | Typical way of life or manner of living characteristic of an individual or group. |
| 40 | ((life* or living) adj3 (change* or skill* or style)).ti,ab,kf. | Keyword | N/A |
| 41 | Budgets/ | MeSH Heading | Detailed financial plans for carrying out specific activities for a certain period of time. They include proposed income and expenditures. |
| 42 | Financial Management/ | MeSH Heading | The obtaining and management of funds for institutional needs and responsibility for fiscal affairs. |
| 43 | ((budget* or financ* or money) adj3 (manag* or responsib* or skill*)).ti,ab,kf. | Keyword | N/A |
| 44 | Hobbies/ | MeSH Heading | Leisure activities engaged in for pleasure. |
| 45 | Leisure Activities/ | MeSH Heading | Voluntary use of free time for activities outside the daily routine. |
| 46 | Recreation/ | MeSH Heading | Activity engaged in for pleasure. |
| 47 | (play* or hobby or hobbies or leisure* or recreation*).ti,ab,kf. | Keyword | N/A |
| 48 | Psychosocial Functioning/ | MeSH Heading | Level of and variations in social and emotional activity or behaviors. |
| 49 | ((psychosocial or psycho social) adj3 function*).ti,ab,kf. | Keyword | N/A |
| 50 | Functional Status/ | MeSH Heading | Ability regarding basic physical and cognitive activities such as walking or reaching, focusing attention, and communicating, as well as the routine activities of daily living, including eating, bathing, dressing, transferring, and toileting; and life situations such as school or play for children and, for adults, work outside the home or maintaining a household. |
| 51 | (functional adj3 (abilit* or capacit* or status)).ti,ab,kf. | Keyword | N/A |
| 52 | or/5-51 |  |  |
| 53 | (approach* or group* or intervention* or program* or rehabilit* or service*).ti,ab,kf. | Keyword | Not applicable |
| 54 | Program Development/ | MeSH Heading | The process of formulating, improving, and expanding educational, managerial, or service-oriented work plans (excluding computer program development). |
| 55 | Program Evaluation/ | MeSH Heading | Studies designed to assess the efficacy of programs. They may include the evaluation of cost-effectiveness, the extent to which objectives are met, or impact. |
| 56 | Psychiatric Rehabilitation/ | MeSH Heading | Specialty field that promotes recovery, community functioning, and increased well-being of individuals diagnosed with mental disorders that impair their ability to live meaningful lives. |
| 57 | Psychosocial Intervention/ | MeSH Heading | Psychological and social interventions by providers including psychologists, psychiatrists, social workers, counselors/therapists, primary care and other nonpsychiatric physicians, nurses, physical and occupational therapists, religious leaders, lay and peer providers, paraprofessionals and caregivers, and automated providers (e.g. internet/audio/video-delivered interventions). Combinations of provider options are sometimes used. |
| 58 | Rehabilitation/ | MeSH Heading | Restoration of human functions to the maximum degree possible in a person or persons suffering from disease or injury. |
| 59 | or/53-58 |  |  |
| 60 | 4 and 52 and 59 |  |  |

## Embase and Emcare

| Line | Search term | Search type | Scope |
| --- | --- | --- | --- |
| 1 | borderline state/ | Subject Heading | A personality disorder marked by a pervasive instability of mood, self−image or sense of self, and interpersonal relationships; impulsive and self−damaging acts are common, as are uncontrolled anger, fears of abandonment, chronic feelings of emptiness, recurrent self−mutilating behavior and suicide threats, and transient, stress−induced periods of paranoia and dissociation. |
| 2 | ((borderline or emotionally unstable) adj1 personality disorder*).ti,ab,kf. | Keyword | N/A |
| 3 | ("F60.3" or "F60.30" or "F60.31").ti,ab,kf. | Keyword | N/A |
| 4 | or/1-3 |  |  |
| 5 | ((self or personal) adj3 (appearance or care or hygiene or manage*)).ti,ab,kf. | Keyword | N/A |
| 6 | self care/ | Subject Heading | Not available |
| 7 | laundry/ | Subject Heading | Not available |
| 8 | (laundry or laundering).ti,ab,kf. | Keyword | N/A |
| 9 | feeding behavior/ | Subject Heading | Not available |
| 10 | eating habit/ | Subject Heading | Not available |
| 11 | ((diet* or eat* or feed* or food) adj3 (behav* or choice* or habit*)).ti,ab,kf. | Keyword | N/A |
| 13 | sedentary lifestyle/ | Subject Heading | Not available |
| 14 | (physical adj3 (activit* or condition or exercise* or fitness or wellbeing or well being)).ti,ab,kf. | Keyword | N/A |
| 15 | (sedentary adj3 (behav* or life*)).ti,ab,kf. | Keyword | N/A |
| 16 | sleep hygiene/ | Subject Heading | Not available |
| 17 | (sleep adj3 (habit* or hygien* or routine* or schedule*)).ti,ab,kf. | Keyword | N/A |
| 18 | attitude to health/ | Subject Heading | Not available |
| 19 | health behavior/ | Subject Heading | Not available |
| 20 | health literacy/ | Subject Heading | Not available |
| 21 | health status/ | Subject Heading | Not available |
| 22 | (health adj3 (attitude* or behav* or knowledge* or literacy or maint* or state or status)).ti,ab,kf. | Keyword | N/A |
| 23 | medication compliance/ | Subject Heading | Not available |
| 24 | (medication* adj3 (adherence or compliance or manag*)).ti,ab,kf. | Keyword | N/A |
| 25 | housing/ | Subject Heading | Not available |
| 26 | household/ | Subject Heading | Not available |
| 27 | ((domestic or home or house* or housing) adj3 (activit* or chore* or clean* or duty or duties or keeping or maint* or skill* or task*)).ti,ab,kf. | Keyword | N/A |
| 28 | cooking/ | Subject Heading | Not available |
| 30 | ((cook* or food or grocery or groceries or meal) adj3 (plan* or prep* or shop* or skill*)).ti,ab,kf. | Keyword | N/A |
| 31 | time management/ | Subject Heading | Not available |
| 32 | (time adj3 (manag* or organis* or organiz* or priorit* or structur*)).ti,ab,kf. | Keyword | N/A |
| 33 | independent living/ | Subject Heading | Not available |
| 34 | independent living.ti,ab,kf. | Keyword | N/A |
| 36 | (fatigue adj3 manag*).ti,ab,kf. | Keyword | N/A |
| 39 | ((activit* adj3 daily living) or ADL).ti,ab,kf. | Keyword | N/A |
| 40 | ((daily or everyday) adj3 (activit* or function* or habit* or life or living or routine* or schedul* or task*)).ti,ab,kf. | Keyword | N/A |
| 41 | lifestyle/ | Subject Heading | Not available |
| 42 | lifestyle modification/ | Subject Heading | Not available |
| 43 | ((life or living) adj3 (skill* or style)).ti,ab,kf. | Keyword | N/A |
| 44 | budget/ | Subject Heading | Not available |
| 45 | financial management/ | Subject Heading | Not available |
| 46 | ((budget* or financ* or money) adj3 (manag* or responsib* or skill*)).ti,ab,kf. | Keyword | N/A |
| 47 | leisure/ | Subject Heading | Not available |
| 48 | recreation/ | Subject Heading | Not available |
| 49 | (play* or hobby or hobbies or leisure* or recreation*).ti,ab,kf. | Keyword | N/A |
| 50 | ((psychosocial or psycho social) adj3 function*).ti,ab,kf. | Keyword | N/A |
| 51 | functional status/ | Subject Heading | Not available |
| 52 | (functional adj3 (abilit* or capacit* or status)).ti,ab,kf. | Keyword | N/A |
| 53 | or/5-52 |  |  |
| 54 | (approach* or group* or intervention* or program* or rehabilit* or service*).ti,ab,kf. | Keyword | N/A |
| 55 | program development/ | Subject Heading | Not available |
| 56 | program evaluation/ | Subject Heading | Not available |
| 57 | psychosocial rehabilitation/ | Subject Heading | Not available |
| 58 | psychosocial intervention/ | Subject Heading | Not available |
| 59 | rehabilitation/ | Subject Heading | The restoration of normal form and function after injury or illness. |
| 60 | or/54-59 |  |  |
| 61 | 4 and 53 and 60 |  |  |

## APA PsycInfo

| Line | Search term | Search type | Scope |
| --- | --- | --- | --- |
| 1 | Borderline Personality Disorder/ | Subject Heading | Personality disorder with maladaptive patterns of behavior characterized by impulsive and unpredictable actions, mood instability, and unstable interpersonal relationships. |
| 2 | ((borderline or emotionally unstable) adj1 personality disorder*).ti,ab. | Keyword | N/A |
| 3 | ("F60.3" or "F60.30" or "F60.31").ti,ab. | Keyword | N/A |
| 4 | or/1-3 |  |  |
| 5 | ((self or personal) adj3 (appearance or care or hygiene or manage*)).ti,ab. | Keyword | N/A |
| 6 | Self-Care/ | Subject Heading | Activities or behaviors that enhance mental, emotional, and/or physical health and well-being. |
| 7 | (laundry or laundering).ti,ab. | Keyword | N/A |
| 8 | Eating Behavior/ | Subject Heading | Not available |
| 9 | ((diet* or eat* or feed* or food) adj3 (behav* or choice* or habit*)).ti,ab. | Keyword | N/A |
| 10 | Physical Fitness/ | Subject Heading | None available |
| 11 | Sedentary Behavior/ | Subject Heading | Signifies a persistent lack of physical activity, which can lead to anxiety, cardiovascular disease, obesity and other related disorders. |
| 12 | (physical adj3 (activit* or condition or exercise* or fitness or wellbeing or well being)).ti,ab. | Keyword | N/A |
| 13 | (sedentary adj3 (behav* or life*)).ti,ab. | Keyword | N/A |
| 14 | Sleep Quality/ | Subject Heading | Subjective and objective factors in sleep satisfaction, duration, and adequacy, not specific to an individual sleep disorder. |
| 15 | (sleep adj3 (habit* or hygiene or quality or routine* or schedule*)).ti,ab. | Keyword | N/A |
| 16 | Health Attitudes/ | Subject Heading | Beliefs or opinions about health, disease, health care services, or other health-related issues. |
| 17 | Health Behavior/ | Subject Heading | Individual lifestyle and behavior which may or may not enhance or maintain good health. |
| 18 | Health Literacy/ | Subject Heading | The ability to understand health information and to use that information to make appropriate health decisions. |
| 19 | Health Status/ | Subject Heading | The degree and indicators of health of an individual, group, or population, assessed by self-report, surveys, or other measures. |
| 20 | (health adj3 (attitude* or behav* or knowledge* or literacy or maint* or state or status)).ti,ab. | Keyword | N/A |
| 21 | Treatment Compliance/ | Subject Heading | Adherence by a patient or client to professional advice or a systematic plan of treatment. |
| 22 | (medication* adj3 (adherence or compliance or manag*)).ti,ab. | Keyword | N/A |
| 23 | Housing/ | Subject Heading | Not available |
| 24 | Household Management/ | Subject Heading | Activities carried out for the regular maintenance of home and personal belongings. |
| 25 | ((domestic or home or house* or housing) adj3 (activit* or chore* or clean* or duty or duties or keeping or maint* or manage* or skill* or task*)).ti,ab. | Keyword | N/A |
| 26 | Food Preparation/ | Subject Heading | Preparation of food for consumption, including but not limited to cooking. |
| 27 | Mealtimes/ | Subject Heading | None available |
| 28 | ((cook* or food or grocery or groceries or meal) adj3 (plan* or prep* or shop* or skill*)).ti,ab. | Keyword | N/A |
| 29 | Time Management/ | Subject Heading | Not available |
| 30 | (time adj3 (manag* or organis* or organiz* or priorit* or structur*)).ti,ab. | Keyword | N/A |
| 31 | Self-Care Skills/ | Subject Heading | Skills such as personal hygiene, feeding, independent housekeeping, public transportation use, which are often taught in rehabilitation programs for persons with mental, physical, or emotional handicaps. |
| 32 | independent living.ti,ab. | Keyword | N/A |
| 33 | Fatigue/ | Subject Heading | A usually transient state of discomfort and loss of efficiency as a normal reaction to emotional strain, physical exertion, boredom, or lack of rest. |
| 34 | (fatigue adj3 manag*).ti,ab. | Keyword | N/A |
| 35 | "Activities of Daily Living"/ | Subject Heading | Basic personal care skills such as eating, bathing, dressing, and other personal hygienic skills used to measure functional ability in older adults and individuals with emotional and physical disabilities. |
| 36 | Habits/ | Subject Heading | None available |
| 37 | ((activit* adj3 daily living) or ADL).ti,ab. | Keyword | N/A |
| 38 | ((daily or everyday) adj3 (activit* or function* or habit* or life or living or routine* or schedul* or task*)).ti,ab. | Keyword | N/A |
| 39 | Lifestyle/ | Subject Heading | Typical way of life or manner of living characteristic of an individual or groups. |
| 40 | Lifestyle Changes/ | Subject Heading | Not available |
| 41 | ((life* or living) adj3 (change* or skill* or style)).ti,ab. | Keyword | N/A |
| 42 | Budgets/ | Subject Heading | Not available |
| 43 | Finance/ | Subject Heading | The management of money and assets. It also includes money or credit to make purchases. |
| 44 | ((budget* or financ* or money) adj3 (manag* or responsib* or skill*)).ti,ab. | Keyword | N/A |
| 45 | Hobbies/ | Subject Heading | Not available |
| 46 | Leisure Time/ | Subject Heading | Not available |
| 47 | Recreation/ | Subject Heading | Not available |
| 48 | (play* or hobby or hobbies or leisure* or recreation*).ti,ab. | Keyword | N/A |
| 49 | Psychosocial Factors/ | Keyword | Not available |
| 50 | ((psychosocial or psycho social) adj3 function*).ti,ab. | Keyword | N/A |
| 51 | Functional Status/ | Subject Heading | A measure of an individual's ability to independently perform activities of daily living and instrumental activities of daily living, and often used to assess the severity of an individual's disability. |
| 52 | (functional adj3 (abilit* or capacit* or status)).ti,ab. | Keyword | N/A |
| 53 | or/5-52 |  |  |
| 54 | (approach* or group* or intervention* or program* or rehabilit* or service*).ti,ab. | Keyword | Not applicable |
| 55 | Program Development/ | Subject Heading | Formulation and/or implementation of programs in any setting. |
| 56 | Program Evaluation/ | Subject Heading | Assessment of programs in any setting. |
| 57 | Psychosocial Rehabilitation/ | Subject Heading | A range of social, occupational, educational, behavioral, and cognitive interventions focusing on recovery, skill training, and community integration for individuals with mental illness or mental health issues. |
| 58 | Psychosocial Interventions/ | Subject Heading | Broad range of nonpharmacological treatments and therapies that focus on the psychological and social aspects of an individual's health, mental health, and well-being. Psychosocial interventions can be individual or group-based, and include various types of psychotherapy as well as support groups, peer support, and psychoeducation. |
| 59 | Rehabilitation/ | Subject Heading | Interventions designed to restore health or improve abilities necessary for daily functioning. Rehabilitation can be provided due to various conditions, such as acute or chronic disease, disability, injury, substance abuse, or incarceration. |
| 60 | or/54-59 |  |  |
| 61 | 4 and 53 and 60 |  |  |

## CINAHL

| Line | Search term | Search type | Scope |
| --- | --- | --- | --- |
| S1 | (MH "Borderline Personality Disorder") | Subject Heading | A disorder characterized by instability in several areas -- interpersonal relationships, marked shifts in mood, and uncertainty about identity issues -- which causes impairment in social or occupational functioning or subjective distress. |
| S2 | TI ( ((borderline or emotionally unstable) w1 personality disorder*) ) OR AB ( ((borderline or emotionally unstable) w1 personality disorder*) ) | Keyword | N/A |
| S3 | TI ( ("F60.3" or "F60.30" or "F60.31") ) OR AB ( ("F60.3" or "F60.30" or "F60.31") ) | Keyword | N/A |
| S4 | S1 or S2 or S3 |  |  |
| S5 | TI ( ((self or personal) w2 (appearance or care or hygiene or manage*)) ) OR AB ( ((self or personal) w2 (appearance or care or hygiene or manage*)) ) | Keyword | N/A |
| S6 | (MH "Self Care") | Subject Heading | Consumer performance of activities for oneself. |
| S7 | TI ( (laundry or laundering) ) OR AB ( (laundry or laundering) ) | Keyword | N/A |
| S8 | (MH "Eating Behavior") | Subject Heading | Behavioral responses or sequences associated with eating including modes of feeding, rhythmic patterns of eating, and time intervals. |
| S9 | (MH "Food Habits") | Subject Heading | Acquired or learned dietary practices. |
| S10 | TI ( ((diet* or eat* or feed* or food) w2 (behav* or choice* or habit*)) ) OR AB ( ((diet* or eat* or feed* or food) w2 (behav* or choice* or habit*)) ) | Keyword | N/A |
| S11 | (MH "Physical Fitness") | Subject Heading | The ability to perform physical activities, especially exercises, without fatigue or exertion. |
| S12 | (MH "Life Style, Sedentary") | Subject Heading | A pattern of living involving little or no exercise. |
| S13 | TI ( (physical w2 (activit* or condition or exercise* or fitness or well$being)) ) OR AB ( (physical w2 (activit* or condition or exercise* or fitness or wellbeing or "well being")) ) | Keyword | N/A |
| S14 | TI ( (sedentary w2 (behav* or life*)) ) OR AB ( (sedentary w2 (behav* or life*)) ) | Keyword | N/A |
| S15 | (MH "Sleep Hygiene") | Subject Heading | Habits and practices that affect the quality of one's sleep. |
| S16 | TI ( (sleep w2 (habit* or hygiene or routine* or schedule*)) ) OR AB ( (sleep w2 (habit* or hygiene or routine* or schedule*)) ) | Keyword | N/A |
| S17 | (MH "Attitude to Health") | Subject Heading | Attitude toward health and the health care system. |
| S18 | (MH "Health Behavior") | Subject Heading | Actions taken by an individual to maintain or improve health status, such as diet and exercise. |
| S19 | (MH "Health Literacy") | Subject Heading | The ability to understand and process health-related information that is necessary in guiding health behavior and decision-making in attaining health services. |
| S20 | (MH "Health Status") | Subject Heading | The level of health of an individual or group, assessed by either subjective or objective means. |
| S21 | TI ( (health w2 (attitude* or behav* or knowledge* or literacy or maint* or state or status)) ) OR AB ( (health w2 (attitude* or behav* or knowledge* or literacy or maint* or state or status)) ) | Keyword | N/A |
| S22 | (MH "Medication Compliance") | Subject Heading | Following a prescribed or advised drug or chemical agent regimen. Use for discussion of non-compliance also. |
| S23 | TI ( (medication* w2 (adherence or compliance or manag*)) ) OR AB ( (medication* w2 (adherence or compliance or manag*)) ) | Keyword | N/A |
| S24 | (MH "Housing") | Subject Heading | Living spaces used for shelter or lodging. |
| S25 | TI ( ((domestic or home or house* or housing) w2 (activit* or chore* or clean* or duty or duties or keeping or maint* or manage* or skill* or task*)) ) OR AB ( ((domestic or home or house* or housing) w2 (activit* or chore* or clean* or duty or duties or keeping or maint* or manage* or skill* or task*)) ) | Keyword | N/A |
| S26 | (MH "Cooking") | Subject Heading | The use of heat or microwaves to prepare food. |
| S27 | (MH "Meal Preparation") | Subject Heading | The planning, preparing and serving of a meal to one's self or others. |
| S28 | TI ( ((cook* or food or grocery or groceries or meal) w2 (plan* or prep* or shop* or skill*)) ) OR AB ( ((cook* or food or grocery or groceries or meal) w2 (plan* or prep* or shop* or skill*)) ) | Keyword | N/A |
| S29 | (MH "Time Management") | Subject Heading | The process of organizing and planning how to divide time between tasks in order to work efficiently to complete them. |
| S30 | TI ( (time w2 (manag* or organis* or organiz* or priorit* or structur*)) ) OR AB ( (time w2 (manag* or organis* or organiz* or priorit* or structur*)) ) | Keyword | N/A |
| S31 | (MH "Community Living") | Subject Heading | Living in the community as opposed to living in a facility. |
| S32 | TI "independent living" OR AB "independent living" | Keyword | N/A |
| S33 | (MH "Fatigue") | Subject Heading | A state of feeling severely overtired or a lack of energy which can be constant and limiting. |
| S34 | TI (fatigue w2 manag*) OR AB (fatigue w2 manag*) | Keyword | N/A |
| S35 | (MH "Activities of Daily Living") | Subject Heading | The performance of both basic and instrumental human activities involved in self care: dressing, ambulation, eating, etc. |
| S36 | (MH "Habits") | Subject Heading | Repetitive behaviors or actions carried out automatically and without conscious thought. |
| S37 | TI ( ((activit* w2 daily living) or ADL) ) OR AB ( ((activit* w2 daily living) or ADL) ) | Keyword | N/A |
| S38 | TI ( ((daily or everyday) w2 (activit* or function* or habit* or life or living or routine* or schedul* or task*)) ) OR AB ( ((daily or everyday) w2 (activit* or function* or habit* or life or living or routine* or schedul* or task*)) ) | Keyword | N/A |
| S39 | (MH "Life Style") | Subject Heading | Living or existing with a characteristically individual and distinct manner or pattern in doing things. |
| S40 | (MH "Life Style Changes") | Subject Heading | Modification of an individual's own habitual activities, behavior, and thought processes. |
| S41 | TI ( ((life* or living) w2 (change* or skill* or style)) ) OR AB ( ((life* or living) w2 (change* or skill* or style)) ) | Keyword | N/A |
| S42 | (MH "Budgets") | Subject Heading | A planned estimate of income and expenditure for a set period of time. |
| S43 | (MH "Financial Management") | Subject Heading | All aspects of financial decision-making for a person or for a company. |
| S44 | TI ( ((budget* or financ* or money) w2 (manag* or responsib* or skill*)) ) OR AB ( ((budget* or financ* or money) w2 (manag* or responsib* or skill*)) ) | Keyword | N/A |
| S45 | (MH "Hobbies") | Subject Heading | Leisure activities or interests pursued for enjoyment or relaxation. |
| S46 | (MH "Leisure Activities") | Subject Heading | Activities that are performed during one's free time. |
| S47 | (MH "Recreation") | Subject Heading | Activities performed for relaxation, entertainment, diversion or pleasure. |
| S48 | TI ( (play* or hobby or hobbies or leisure* or recreation*) ) OR AB ( (play* or hobby or hobbies or leisure* or recreation*) ) | Keyword | N/A |
| S49 | (MH "Psychosocial Functioning") | Subject Heading | The extent to which an individual is able to engage in normal social, emotional and behavioral activities of daily living. |
| S50 | TI ( ((psychosocial or "psycho social") w2 function*) ) OR AB ( ((psychosocial or "psycho social") w2 function*) ) | Keyword | N/A |
| S51 | (MH "Functional Status") | Subject Heading | An individual's level of abilities and limitations in performing tasks necessary to daily living, leisure activities, vocational pursuits, social interactions, and other required behavior or activities. |
| S52 | TI ( (functional w2 (abilit* or capacit* or status)) ) OR AB ( (functional w2 (abilit* or capacit* or status)) ) | Keyword | N/A |
| S53 | S5 or S6 or S7 or S8 or S9 or S10 or S11 or S12 or S13 or S14 or S15 or S16 or S17 or S18 or S19 or S20 or S21 or S22 or S23 or S24 or S25 or S26 or S27 or S28 or S29 or S30 or S31 or S32 or S33 or S34 or S35 or S36 or S37 or S38 or S39 or S40 or S41 or S42 or S43 or S44 or S45 or S46 or S47 or S48 or S49 or S50 or S51 or S52 |  |  |
| S54 | TI ( (approach* or group* or intervention* or program* or rehabilit* or service*) ) OR AB ( (approach* or group* or intervention* or program* or rehabilit* or service*) ) | Keyword | N/A |
| S55 | (MH "Program Development") | Subject Heading | Process of devising an overarching plan to follow in developing a program. Encompasses activities of planning, implementation, and evaluation. |
| S56 | (MH "Program Evaluation") | Subject Heading | Assessing the results or outcome of a program in relation to stated criteria or objectives. |
| S57 | (MH "Rehabilitation, Psychosocial") | Subject Heading | Care provided to improve emotional, social, and intellectual skills in order to live and work successfully. |
| S58 | (MH "Psychosocial Intervention") | Subject Heading | A form of psychotherapy and counseling that utilizes both social and psychological actions and techniques to improve health and functioning. |
| S59 | (MH "Rehabilitation") | Subject Heading | Care provided to restore the ability to live and work as normally as possible following a disabling injury or illness. |
| S60 | S54 or S55 or S56 or S57 or S58 or S59 |  |  |
| S61 | S4 and S53 and S60 |  |  |

## ProQuest and ASSIA

| Line | Search term |
| --- | --- |
|  | TI,AB,SU((borderline OR "emotionally unstable") N/1 personality disorder*) OR ("F60.3" or "F60.30" or "F60.31") |
| AND | TI,AB,SU((self OR personal) N/2 (appearance OR care OR hygiene OR manage*)) OR TI,AB,SU(laundry OR laundering) OR TI,AB,SU((diet* or eat* or feed* or food) N/2 (behav* or choice* or habit*)) OR TI,AB,SU(physical N/2 (activit* or condition or exercise* or fitness or "well being")) OR TI,AB,SU(sedentary N/2 (behav* or "life style")) OR TI,AB,SU(sleep N/2 (habit* or hygiene or routine* or schedule*)) OR TI,AB,SU(health N/2 (attitude* or behav* or knowledge* or literacy or maint* or state or status)) OR TI,AB,SU(medication* N/2 (adherence or compliance or manag*)) OR TI,AB,SU((domestic or home or house* or housing) N/2 (activit* or chore* or clean* or duty or duties or keeping or maint* or manage* or skill* or task*)) OR TI,AB,SU((cook* or food or grocery or groceries or meal) N/2 (plan* or prep* or shop* or skill*)) OR TI,AB,SU(time N/2 (manag* or organis* or organiz* or priorit* or structur*)) OR TI,AB,SU(independent living) OR TI,AB,SU(fatigue N/2 manag*) OR TI,AB,SU((activit* N/2 daily living) or ADL) OR TI,AB,SU((daily or everyday) N/2 (activit* or function* or habit* or life or living or routine* or schedul* or task*)) OR TI,AB,SU((life* or living) N/2 (change* or skill* or style)) OR TI,AB,SU((budget* or financ* or money) N/2 (manag* or responsib* or skill*)) OR TI,AB,SU(play* or hobby or hobbies or leisure* or recreation*) OR TI,AB,SU((psychosocial or "psycho social") N/2 function*) OR TI,AB,SU(functional N/2 (abilit* or capacit* or status)) |
| AND | TI,AB,SU(approach* or group* or intervention* or program* or rehabilit* or service*) |

## Scopus

| Line | Search term |
| --- | --- |
|  | ( TITLE-ABS-KEY ( ( ( borderline OR "emotionally unstable" ) W/1 "personality disorder*" ) OR ( "f60.3" OR "f60.30" OR "f60.31" ) ) |
| AND | ( TITLE-ABS-KEY ( ( self OR personal ) W/2 ( appearance OR care OR hygiene OR manage* ) ) ) OR ( TITLE-ABS-KEY ( laundry OR laundering ) ) OR ( TITLE-ABS-KEY ( ( ( diet* OR eat* OR feed* OR food ) W/2 ( behav* OR choice* OR habit* ) ) ) ) OR ( TITLE-ABS-KEY ( ( sleep W/2 ( habit* OR hygiene OR routine* OR schedule* ) ) ) ) OR ( TITLE-ABS-KEY ( ( health W/2 ( attitude* OR behav* OR knowledge* OR literacy OR maint* OR state OR status ) ) ) ) OR ( TITLE-ABS-KEY ( ( medication* W/2 ( adherence OR compliance OR manag* ) ) ) ) OR ( TITLE-ABS-KEY ( ( ( domestic OR home OR house* OR housing ) W/2 ( activit* OR chore* OR clean* OR duty OR duties OR keeping OR maint* OR manage* OR skill* OR task* ) ) ) ) OR ( TITLE-ABS-KEY ( ( ( cook* OR food OR grocery OR groceries OR meal ) W/2 ( plan* OR prep* OR shop* OR skill* ) ) ) ) OR ( TITLE-ABS-KEY ( ( time W/2 ( manag* OR organis* OR organiz* OR priorit* OR structur* ) ) ) ) OR ( TITLE-ABS-KEY ( ( fatigue W/2 manag* ) ) ) OR ( TITLE-ABS-KEY ( "independent living" ) ) OR ( TITLE-ABS-KEY ( ( ( daily OR everyday ) W/2 ( activit* OR function* OR habit* OR life OR living OR routine* OR schedul* OR task* ) ) ) ) OR ( TITLE-ABS-KEY ( ( ( activit* W/2 "daily living" ) OR adl ) ) ) OR ( TITLE-ABS-KEY ( ( ( life* OR living ) W/2 ( change* OR skill* OR style ) ) ) ) OR ( TITLE-ABS-KEY ( ( ( budget* OR financ* OR money ) W/2 ( manag* OR responsib* OR skill* ) ) ) ) OR ( TITLE-ABS-KEY ( ( play* OR hobby OR hobbies OR leisure* OR recreation* ) ) ) OR ( TITLE-ABS-KEY ( ( psycho$social W/2 function* ) ) ) OR ( TITLE-ABS-KEY ( ( ( psychosocial OR psycho-social OR "psycho social" ) W/2 function* ) ) ) OR ( TITLE-ABS-KEY ( "functional status" ) ) |
| AND | TITLE-ABS-KEY ( ( approach* OR group* OR intervention* OR program* OR rehabilit* OR service* ) ) ) |

# Grey literature search

## Google and Google Scholar search terms

The search terms below were conducted in August 2025:

“borderline personality disorder”; “psychosocial”; “daily living”; “program”; “group”

## BPD organisation websites

All websites in the table below were searched in August 2025.

* organisation was contacted in February 2024.

^ organisation responded.

| Organisation | Country | Website |
| --- | --- | --- |
| Adelaide PHN * | Australia | https://adelaidephn.com.au/ |
| Amanda Smith Treatment and Consultation | United States | https://www.hopeforbpd.com/ |
| American Psychiatric Association *^ | United States | https://www.psychiatry.org/ |
| Australian BPD Foundation *^ | Australia | https://www.bpdfoundation.org.au/ |
| Bedrock Recovery Centre | United States | <https://bedrockrecoverycenter.com/primary-mental-health-disorders-we-treat/personality-disorders/> |
| Borderline Personality Disorder Collaborative (BPD Co) *^ | Australia | https://www.sahealth.sa.gov.au/ |
| Borderline Personality Disorder Resource Center *^ | United States | <https://www.nyp.org/bpdresourcecenter> |
| Borderline Personality Disorder Society of BC | Canada | <https://www.bpdbc.ca/> |
| Borderline Personality Disorder Support Services in South Australia | Australia | <https://bpdsa.com.au/support-services/individual-group-programs/> |
| Borderline Support UK CIC * | United Kingdom | <https://borderlinesupport.org.uk/> |
| BPD Community * | Australia | <https://www.bpdcommunity.com.au/working-with-bpd/working-with-bpd1> |
| BPD Global | Unknown | <https://www.bpdglobal.com/about-us/> |
| BPD World * | United Kingdom | <https://bpdworld.org/> |
| Bridges to Recovery * | United States | <https://www.bridgestorecovery.com/our-program/> |
| BrightQuest Treatment Centers | United States | <https://www.brightquest.com/borderline-personality-disorder/> |
| Brightshores Health System * | Canada | <https://www.brightshores.ca/mental-health-addiction-services/> |
| Canberra Health Services * | Australia | https://www.canberrahealthservices.act.gov.au/services-and-clinics/services/adult-mental-health-day-stay-service |
| Charlie Waller Memorial Trust, The * | United Kingdom | <https://charliewaller.org/> |
| Clearview Outpatient | United States | <https://clearviewoutpatient.com/treatment/what-we-treat/bpd-and-other-personality-disorders/> |
| Country SA PHN * | Australia | https://www.countrysaphn.com.au/ |
| Department of Health and Aged Care *^ | Australia | <https://www.health.gov.au/> |
| Emotions Matter *^ | United States | <https://emotionsmatterbpd.org/> |
| European Society for the Study of Personality Disorders (ESSPD) * | Nether-lands | <https://www.esspd.eu/> |
| Florida Borderline Personality Disorder Association * | United States | https://fbpda.org/getting-help/treating-bpd/#more-14 |
| International Society for the Study of Personality Disorders (ISSPD) | Denmark | <https://www.isspd.com/> |
| King's Health Partners | United Kingdom | <https://www.kingshealthpartners.org/our-work> |
| Life Adjustment Team * | United States | <https://www.lifeadjustmentteam.com/psychosocial-rehabilitation-in-los-angeles/> |
| McLean Hospital *^ | United States | <https://www.mcleanhospital.org/treatment/bpd-programs> |
| Melbourne Clinic | Australia | <https://themelbourneclinic.com.au/application/files/9515/2687/8715/TMC_Day_Programs.pdf> |
| Mental Health America * | United States | <https://mhanational.org/> |
| Mental Health and Wellbeing Commission * | Australia | <https://www.mhwc.vic.gov.au/> |
| Mental Health Commission, The * | Australia | <https://www.mhc.wa.gov.au/> |
| Mental Health Council of Tasmania *^ | Australia | <https://mhct.org/> |
| Mind *^ | United Kingdom | <https://www.mind.org.uk/> |
| Mind Australia | Australia | <https://www.mindaustralia.org.au/> |
| National Alliance on Mental Illness *^ | United States | <https://www.nami.org/home> |
| National Education Alliance for Borderline Personality Disorder (NEA BPD) * | Australia | <https://www.bpdaustralia.org/> |
| National Mental Health Commission * | Australia | <https://www.mentalhealthcommission.gov.au/> |
| New England Personality Disorders Association | United States | <https://www.nepda.org/> |
| North American Society for the Study of Personality Disorders (NASSPD) | United States | <https://nasspd.org/> |
| Northern Territory Mental Health Commission * | Australia | https://www.ntmhc.org.au/ |
| NSW Mental Health Commission *^ | Australia | <https://www.nswmentalhealthcommission.com.au/content/contact-us> |
| Office for Mental Health and Wellbeing | Australia | <https://www.act.gov.au/directorates-and-agencies/health-and-community-services-directorate/office-for-mental-health-and-wellbeing> |
| Ohio Recovery Centre * | United States | <https://www.recoveryohio.org/personality-disorders/> |
| Orygen * | Australia | <https://www.orygen.org.au/> |
| Pacific Teen Treatment * | United States | <https://pacificteentreatment.com/personality-disorders/borderline-personality-disorder/> |
| Personality Disorder Awareness Network (PDAN) * | United States | <https://pdan.org/> |
| Primary Health Networks * | Australia | <https://www.health.gov.au/our-work/phn> |
| Project Air * | Australia | <https://www.uow.edu.au/project-air/> |
| Queensland Mental Health Commission *^ | Australia | <https://www.qmhc.qld.gov.au/> |
| ReachOut Australia *^ | Australia | <https://au.reachout.com/> |
| Red Fish Healing Centre * | Canada | http://www.bcmhsus.ca/our-services/provincial-integrated-mental-health-addiction-programs/red-fish-healing-centre |
| Resource Residential Treatment Facility | United States | <https://www.resourcetreatmentcenter.com/borderline-personality/> |
| SA Mental Health Commission (SAMHC) *^ | Australia | <https://samentalhealthcommission.com.au/> |
| San Jose Behavioral Health | United States | <https://www.sanjosebh.com/disorders/borderline-personality/> |
| SANE Australia *^ | Australia | <https://www.sane.org/> |
| Sashbear Foundation, The *^ | Canada | <https://sashbear.org/> |
| Sierra Tucson | United States | <https://www.sierratucson.com/disorders/bpd/> |
| Skyland Trail * | United States | <https://www.skylandtrail.org/our-programs/what-we-treat/borderline-personality-disorder/adult-borderline-personality-disorder/> |
| Spectrum | Australia | <https://www.spectrumbpd.com.au/> |
| TARA *^ | United States | <https://www.tara4bpd.org/> |
| Utah Trauma & Addiction Centers * | United States | <https://utahaddictioncenters.com/borderline-personality-disorder/> |
| ZwavelStream Clinic | South Africa | <https://zwavelstreamclinic.co.za/personality-disorders-treatment/> |

## Email template

Subject: Programs addressing the daily living functioning of people with BPD

Hello,

I’m doing a scoping review of programs, approaches and groups that address broader domains of daily living functioning of people with borderline personality disorder (BPD).

Your organisation came up during my grey literature search.

I’m interested in programs that address broader domains of daily living functioning that are not necessarily the focus of psychotherapy. For example, functional domains such as self-care, household maintenance, meal preparation and establishing a meaningful routine.

Are you aware of any programs like this in [location]?

Notably, I will not be sending you a follow up email to this one. Thank you for your time.

With regards,

Dillon Tepper

PhD Candidate - Occupational Therapist

# Measures used to describe changes in functioning

| **Measure used to evaluate functioning** | **Valid** | **Proof of validity** | **Record** | **Program** |
| --- | --- | --- | --- | --- |
| Actigraph GT9X (wrist-worn accelerometer) | No | Suau, Q., Bianchini, E., Bellier, A., Chardon, M., Milane, T., Hansen, C., & Vuillerme, N. (2024). Current knowledge about ActiGraph GT9X link activity monitor accuracy and validity in measuring steps and energy expenditure: a systematic review. Sensors, 24(3), 825. | de Girolamo (2024) | Structured physical activity intervention |
| Adaptive Behavior Scale (AAMD) | Yes | Spreat, S. (1982). The AAMD Adaptive Behavior Scale: A psychometric review. Journal of School Psychology. | Nelson (1987) | Vocational Readiness and Independent Living skills (VRIL) training program |
| Adherence exercise sessions | No | Not applicable/bespoke | St-Amour (2024) | Structured physical exercise |
| Alcohol Use Disorder Identification Test (AUDIT) | Yes | Schøler, P. N., Andersen, M. B., Andersen, K., Becker, U., Thiele, M., & Nielsen, A. S. (2025). Validation of the alcohol use disorders identification test in a Danish hospital setting. Substance Abuse Treatment, Prevention, and Policy, 20(1), 7. | St-Amour (2024) | Structured physical exercise |
| Assessment of Independent Living Skills (AILS) | No | Bespoke (in record) | McAlpine (1991) | Sharing Life in the Community Program (SLIC) |
| Beck Anxiety Inventory (BAI) | Yes | Fydrich, T., Dowdall, D., & Chambless, D. L. (1992). Reliability and validity of the Beck Anxiety Inventory. Journal of anxiety disorders, 6(1), 55-61. | Sayk (2025) | Imagery Rehearsal Therapy |
| Beck Depression Inventory (BDI) | Yes | Richter, P., Werner, J., Heerlein, A., Kraus, A., & Sauer, H. (1998). On the validity of the Beck Depression Inventory: A review. Psychopathology, 31(3), 160-168. | Bromundt (2013) | Light Therapy |
| Beck Depression Inventory short form (BDI-13) | Yes | Pessoa, T. E., Paiva, T. T., Santos, I. L. S., Pimentel, C. E., & Mariano, T. E. (2022). Structural analysis of the Beck Depression Inventory short version (BDI-13). Revista Interamericana de Psicología/Interamerican Journal of Psychology, 56(2), e1515-e1515. | de Girolamo (2024) | Structured physical activity intervention |
| Beck Depression Inventory-II (BDI-II) | Yes | García-Batista, Z. E., Guerra-Peña, K., Cano-Vindel, A., Herrera-Martínez, S. X., & Medrano, L. A. (2018). Validity and reliability of the Beck Depression Inventory (BDI-II) in general and hospital population of Dominican Republic. PloS one, 13(6), e0199750. | Sayk (2025) | Imagery Rehearsal Therapy |
| Beck Depression Inventory-Short Form (BDI-SF) | Yes | Furlanetto, L. M., Mendlowicz, M. V., & Bueno, J. R. (2005). The validity of the Beck Depression Inventory-Short Form as a screening and diagnostic instrument for moderate and severe depression in medical inpatients. Journal of affective disorders, 86(1), 87-91. | St-Amour (2024) | Structured physical exercise |
| Beck’s Hopelessness Scale (BHS) | Yes | Kocalevent, R. D., Finck, C., Pérez-Trujillo, M., Sautier, L., Zill, J., & Hinz, A. (2017). Standardization of the Beck Hopelessness Scale in the general population. Journal of mental health, 26(6), 516-522. | Gabarda-Blasco (2024) | Adventure Therapy |
| Beery Test of Visual-Motor Integration (VMI) | Yes | Mao, H. F., Li, W., & Lo, J. L. (1999). Construct validity of Beery's developmental test of visual-motor integration for Taiwanese children. The Occupational Therapy Journal of Research, 19(4), 241-257. | Nelson (1987) | Vocational Readiness and Independent Living skills (VRIL) training program |
| Behavior and Symptom Identification Scale (BASIS-32) | Yes | Eisen, S. V., Wilcox, M., Leff, H. S., Schaefer, E., & Culhane, M. A. (1999). Assessing behavioral health outcomes in outpatient programs: Reliability and validity of the BASIS-32. The Journal of Behavioral Health Services & Research, 26(1), 5-17. | Lee (2014) | Prevention and Recovery Centre (PARC) |
| Behavioural Interview (with patient and family) | No | Not applicable/bespoke | Ortega (2004) | Functional behaviour analysis and treatment |
| Behavioural change observations | No | Not applicable/bespoke | Clark (2006) | Community Living Skills Course |
| Behavioural Regulation in Exercise Questionnaire-3 (BREQ-3) | Yes | Cavicchiolo, E., Sibilio, M., Lucidi, F., Cozzolino, M., Chirico, A., Girelli, L., ... & Alivernini, F. (2022). The psychometric properties of the behavioural regulation in exercise questionnaire (BREQ-3): factorial structure, invariance and validity in the Italian context. International journal of environmental research and public health, 19(4), 1937. | de Girolamo (2024) | Structured physical activity intervention |
| Bi-monthly Task Behavior Checklist | No | Not applicable/bespoke | Nelson (1987) | Vocational Readiness and Independent Living skills (VRIL) training program |
| Biological parameters | Yes | Standardised | Brogan (2020) | Vital Mind Reset (VMR) online program |
|  |  |  | Bromundt (2013) | Light Therapy |
|  |  |  | de Girolamo (2024) | Structured physical activity intervention |
|  |  |  | Gabarda-Blasco (2024) | Adventure Therapy |
| Borderline Evaluation of Severity Over Time (BEST) | Yes | Pfohl, B., Blum, N., St. John, D., McCormick, B., Allen, J., & Black, D. W. (2009). Reliability and validity of the Borderline Evaluation of Severity Over Time (BEST): A self-rated scale to measure severity and change in persons with borderline personality disorder. Journal of personality disorders, 23(3), 281-293. | Torres-Soto (2021) | Day Hospital treatment program |
| Borderline Personality Inventory (BPI) | Yes | Leichsenring, F. (1999). Development and first results of the Borderline Personality Inventory: A self-report instrument for assessing borderline personality organization. Journal of personality assessment, 73(1), 45-63. | Bromundt (2013) | Light Therapy |
| Borderline Symptom List (BSL-95) | Yes | Bohus, M., Limberger, M. F., Frank, U., Chapman, A. L., Kühler, T., & Stieglitz, R. D. (2007). Psychometric properties of the borderline symptom list (BSL). Psychopathology, 40(2), 126-132. | Bromundt (2013) | Light Therapy |
| Borderline Symptoms List-23 (BSL-23) | Yes | Soler, J., Vega, D., Feliu-Soler, A., Trujols, J., Soto, Á., Elices, M., ... & Pascual, J. C. (2013). Validation of the Spanish version of the Borderline Symptom List, short form (BSL-23). BMC psychiatry, 13(1), 1317. | St-Amour (2024) | Structured physical exercise |
| Brief Disability Questionnaire (BDQ) | Yes | Bourke, J. A., Nichols-Dunsmuir, A., Begg, A., Dong, H., & Schluter, P. J. (2021). Measuring disability: An agreement study between two disability measures. Disability and Health Journal, 14(2), 100995. | Scanlan (2017) | Hospital to Home (H2H) |
| Bruininks-Oseretsky Test of Motor Proficiency (BOTMP) | Yes | Deitz, J. C., Kartin, D., & Kopp, K. (2007). Review of the Bruininks-Oseretsky test of motor proficiency, (BOT-2). Physical & occupational therapy in pediatrics, 27(4), 87-102. | Nelson (1987) | Vocational Readiness and Independent Living skills (VRIL) training program |
| Canadian Occupational Performance Measure (COPM) | Yes | Carswell, A., McColl, M. A., Baptiste, S., Law, M., Polatajko, H., & Pollock, N. (2004). The Canadian Occupational Performance Measure: a research and clinical literature review. Canadian journal of occupational therapy, 71(4), 210-222. | Lima (2006) | Occupational Therapy |
| Cannabis Abuse Screening Test (CAST) | Yes | Legleye, S., Karila, L., Beck, F., & Reynaud, M. (2007). Validation of the CAST, a general population Cannabis Abuse Screening Test. Journal of substance use, 12(4), 233-242. | St-Amour (2024) | Structured physical exercise |
| Childhood Trauma Questionnaire (CTQ) | Yes | Bernstein, D. P., Ahluvalia, T., Pogge, D., & Handelsman, L. (1997). Validity of the Childhood Trauma Questionnaire in an adolescent psychiatric population. Journal of the American Academy of Child & Adolescent Psychiatry, 36(3), 340-348. | Sayk (2025) | Imagery Rehearsal Therapy |
| Client Satisfaction Questionnaire (CSQ-8) | Yes | Attkisson, C. C., & Zwick, R. (1982). The Client Satisfaction Questionnaire: Psychometric properties and correlations with service utilization and psychotherapy outcome. Evaluation and program planning, 5(3), 233-237. | Scanlan (2017) | Hospital to Home (H2H) |
| Clinical Global Impressions (CGI) Scale | No | Worden, B. (2024). A call for more careful use of the Clinical Global Impression (CGI) rating as a measure of psychopathology and outcome. Psychological Reports, 00332941241301344. | Sayk (2025) | Imagery Rehearsal Therapy |
| Comprehensive Behavioral Task Checklist | No | Stage, S. A., Cheney, D., Walker, B., & LaRocque, M. (2002). A preliminary discriminant and convergent validity study of the teacher functional behavioral assessment checklist. School Psychology Review, 31(1), 71-93. | Nelson (1987) | Vocational Readiness and Independent Living skills (VRIL) training program |
| Dietary compliance | No | Not applicable/bespoke | Brogan (2020) | Vital Mind Reset (VMR) online program |
| Difficulties in Emotion Regulation Scale (DERS) | Yes | Victor, S. E., & Klonsky, E. D. (2016). Validation of a brief version of the difficulties in emotion regulation scale (DERS-18) in five samples. Journal of psychopathology and Behavioral Assessment, 38(4), 582-589. | St-Amour (2024) | Structured physical exercise |
| Ecological Momentary Assessment response rate | No | Not applicable/bespoke | St-Amour (2024) | Structured physical exercise |
| Educational report cards (standard) | No | Not applicable/bespoke | Nelson (1987) | Vocational Readiness and Independent Living skills (VRIL) training program |
| EEG Spectral Power Analysis | Yes | Thatcher, R. W. (2010). Validity and reliability of quantitative electroencephalography. Journal of neurotherapy, 14(2), 122-152. | Sayk (2025) | Imagery Rehearsal Therapy |
| Emotion Regulation Questionnaire (ERQ) | Yes | Spaapen, D. L., Waters, F., Brummer, L., Stopa, L., & Bucks, R. S. (2014). The emotion regulation questionnaire: validation of the ERQ-9 in two community samples. Psychological assessment, 26(1), 46. | Sayk (2025) | Imagery Rehearsal Therapy |
| Evening and morning sleep protocols | No | Not applicable/bespoke | Sayk (2025) | Imagery Rehearsal Therapy |
| Hamilton Depression Scale with Atypical Depression Supplement (SIGH-ADS-SR) | Yes | Trajković, G., Starčević, V., Latas, M., Leštarević, M., Ille, T., Bukumirić, Z., & Marinković, J. (2011). Reliability of the Hamilton Rating Scale for Depression: a meta-analysis over a period of 49 years. Psychiatry research, 189(1), 1-9. | Bromundt (2013) | Light Therapy |
| Health of the Nation Outcome Scale (HoNOS) | No | Brooks, R. (2000). The reliability and validity of the Health of the Nation Outcome Scales: validation in relation to patient derived measures. Australian & New Zealand Journal of Psychiatry, 34(3), 504-511. | Lee (2014) | Prevention and Recovery Centre (PARC) |
|  |  |  | Sekharan (2021) |  |
| Health Promoting Lifestyle Questionnaire (HPLQ) | Yes | Wang, Q., Wang, Y., Xie, X., Wang, Z., Liu, Y., Guo, B., ... & Ge, L. (2023). The measurement properties of existing lifestyle assessment tools are suboptimal: a systematic review. Journal of Public Health, 1-16. | Soleimani (2025) | Coping Skills Training |
| Impact of Event Scale–Revised (IES-R) | Yes | Christianson, S., & Marren, J. (2012). The impact of event scale-revised (IES-R). Medsurg Nurs, 21(5), 321-322. | Sayk (2025) | Imagery Rehearsal Therapy |
| Impulsive Behavior Scale (UPPS-P) | Yes | Verdejo-García, A., Lozano, Ó., Moya, M., Alcázar, M. Á., & Pérez-García, M. (2010). Psychometric properties of a spanish version of the UPPS–P impulsive behavior scale: reliability, validity and association with trait and cognitive impulsivity. Journal of personality assessment, 92(1), 70-77. | de Girolamo (2024) | Structured physical activity intervention |
| Individual Client Checklist | No | Not applicable/bespoke | Nelson (1987) | Vocational Readiness and Independent Living skills (VRIL) training program |
| Individual Recovery Plan (IRP) goal achivement (Short-point Likert scale) | No | Not applicable/bespoke | Sekharan (2021) | Prevention and Recovery Care (PARC) |
| International Personality Disorders Examination (IPDE) | No | Magallón-Neri, E. M., Forns, M., Canalda, G., De La Fuente, J. E., García, R., González, E., ... & Castro-Fornieles, J. (2013). Usefulness of the International Personality Disorder Examination screening questionnaire for borderline and impulsive personality pathology in adolescents. Comprehensive psychiatry, 54(3), 301-308. | Ortega (2004) | Functional behaviour analysis and treatment |
| Inventory of Anxiety Situations and Responses (ISRA) [Translated from Spanish] | Yes | [Cano-Vindel, A., Munoz-Navarro, R., Moretti, L. S., & Medrano, L. A. (2020). Propiedades psicométricas del inventario de situaciones y respuestas de ansiedad breve (ISRA-B). Ansiedad y estrés, 26(2-3), 155-166.](https://recil.ulusofona.pt/server/api/core/bitstreams/b87e8850-0592-43b9-b2bc-901578c9b78d/content) | Ortega (2004) | Functional behaviour analysis and treatment |
| Kessler Psychological Distress Scale (K10) | Yes | Pereira, A., Oliveira, C. A., Bártolo, A., Monteiro, S., Vagos, P., & Jardim, J. (2019). Reliability and factor structure of the 10-item Kessler Psychological Distress Scale (K10) among Portuguese adults. Ciencia & saude coletiva, 24(3), 729-736. | Scanlan (2017) | Hospital to Home (H2H) |
| Key Math Test | Yes | Greenstein, J., & Strain, P. S. (1977). The utility of the Key Math Diagnostic Arithmetic Test for adolescent learning disabled students. Psychology in the Schools, 14(3), 275-282. | Nelson (1987) | Vocational Readiness and Independent Living skills (VRIL) training program |
| Kohlman Evaluation of Living Skills (KELS) | Yes | Burnett, J., Dyer, C. B., & Naik, A. D. (2009). Convergent validation of the Kohlman Evaluation of Living Skills as a screening tool of older adults' ability to live safely and independently in the community. Archives of physical medicine and rehabilitation, 90(11), 1948-1952. | Nelson (1987) | Vocational Readiness and Independent Living skills (VRIL) training program |
| Medication usage | No | Not applicable/bespoke | Brogan (2020) | Vital Mind Reset (VMR) online program |
| Mental Well-Being Questionnaire (MWQ) | Yes | Keyes, C. L., & Magyar-Moe, J. L. (2003). The measurement and utility of adult subjective well-being. | Soleimani (2025) | Coping Skills Training |
| Millon Clinical Multiaxial Inventory – II (MCMI-II) | Yes | Rogers, R., Salekin, R. T., & Sewell, K. W. (1999). Validation of the Millon Clinical Multiaxial Inventory for Axis II disorders: Does it meet the Daubert standard?. Law and Human Behavior, 23(4), 425-443. | Ortega (2004) | Functional behaviour analysis and treatment |
| Model of Human Occupation Screening Tool (MOHOST) | Yes | Kielhofner, G., Fan, C. W., Morley, M., Garnham, M., Heasman, D., Forsyth, K., ... & Taylor, R. R. (2010). A psychometric study of the Model of Human Occupation Screening Tool (MOHOST). Hong Kong Journal of Occupational Therapy, 20(2), 63-70. | Lee (2010) | Occupational Therapy |
| Morningness-Eveningness Questionnaire (MEQ) | Yes | Taillard, J., Philip, P., Chastang, J. F., & Bioulac, B. (2004). Validation of Horne and Ostberg morningness-eveningness questionnaire in a middle-aged population of French workers. Journal of biological rhythms, 19(1), 76-86. | Bromundt (2013) | Light Therapy |
|  |  |  | St-Amour (2024) | Structured physical exercise |
| Motor-Free Visual Perception Test (MFVPT) | Yes | Brown, T., & Peres, L. (2018). A critical review of the motor-free visual perception test—fourth edition (MVPT-4). Journal of Occupational Therapy, Schools, & Early Intervention, 11(2), 229-244. | Nelson (1987) | Vocational Readiness and Independent Living skills (VRIL) training program |
| Multidimensional Mood State Questionnaire (MDBF) | Yes | Steyer, R., Schwenkmezger, P., Notz, P., & Eid, M. (2004). Entwicklung des Mehrdimensionalen Befindlichkeitsfragebogens (MDBF). Primärdatensatz. | Bromundt (2013) | Light Therapy |
| MyFood24 (food diary) | No | Not applicable/bespoke | de Girolamo (2024) | Structured physical activity intervention |
| Nonparametric Circadian Rhythm Analysis (NPCRA) | Yes | Standardised | Bromundt (2013) | Light Therapy |
| Patient Global Impression (PGI) Scale | Yes | Mohebbi, M., Dodd, S., Dean, O. M., & Berk, M. (2018). Patient centric measures for a patient centric era: agreement and convergent between ratings on the Patient Global Impression of Improvement (PGI-I) scale and the Clinical Global Impressions–Improvement (CGI-S) scale in bipolar and major depressive disorder. European Psychiatry, 53, 17-22. | Sayk (2025) | Imagery Rehearsal Therapy |
| Personal Wellness Wheel | No | Gill, K. (2012). New moves: targeting physical and mental well-being in people with mental illness. Health Issues, (108), 18-23. | Scanlan (2017) | Hospital to Home (H2H) |
| Personality Inventory for DSM-5 | Yes | Fossati, A., Krueger, R. F., Markon, K. E., Borroni, S., & Maffei, C. (2013). Reliability and validity of the Personality Inventory for DSM-5 (PID-5) predicting DSM-IV personality disorders and psychopathy in community-dwelling Italian adults. Assessment, 20(6), 689-708. | Torres-Soto (2021) | Day Hospital treatment program |
| Physical activity diary | No | Not applicable/bespoke | de Girolamo (2024) | Structured physical activity intervention |
|  |  |  | Gabarda-Blasco (2024) | Adventure Therapy |
| Physical testing using the ICH E6 (R2) GCP | Yes | Standardised | de Girolamo (2024) | Structured physical activity intervention |
| Pittsburgh Sleep Quality Index (PSQI) | Yes | Lu, T., Li, Y., Xia, P., Zhang, G., & Wu, D. (2014). Analysis on reliability and validity of the Pittsburgh sleep quality index. Chongqing medicine, 260-263. | Bromundt (2013) | Light Therapy |
|  |  |  | Sayk (2025) | Imagery Rehearsal Therapy |
| Plutchik’s Impulsivity Scale | Yes | Alcázar-Córcoles, M. Á., Verdejo, A. J., & Bouso-Sáiz, J. C. (2015). Psychometric Properties of Plutchik’s Impulsivity Scale in Juvenile Spanishspeaking population. Actas Españolas de Psiquiatría, 43(5), 161-169. | Gabarda-Blasco (2024) | Adventure Therapy |
| Polysomnography | Yes | Standardised | Sayk (2025) | Imagery Rehearsal Therapy |
| Positive and Negative Affect Schedule - Short Form (PANAS-SF) | Yes | Karim, J., Weisz, R., & Rehman, S. U. (2011). International positive and negative affect schedule short-form (I-PANAS-SF): Testing for factorial invariance across cultures. Procedia-Social and Behavioral Sciences, 15, 2016-2022. | St-Amour (2024) | Structured physical exercise |
| Positivity scale (P-scale) | Yes | Caprara, G. V., Alessandri, G., Eisenberg, N., Kupfer, A., Steca, P., Caprara, M. G., ... & Abela, J. (2012). The positivity scale. Psychological assessment, 24(3), 701. | de Girolamo (2024) | Structured physical activity intervention |
| Premenstrual Symptom Screening Tool (PSST) | Yes | Steiner, M., Macdougall, M., & Brown, E. (2003). The premenstrual symptoms screening tool (PSST) for clinicians. Archives of women’s mental health, 6(3), 203-209. | de Girolamo (2024) | Structured physical activity intervention |
| Proximal Skin Temperature Monitoring | Yes | Standardised | Bromundt (2013) | Light Therapy |
| Questionnaire | No | Not applicable/bespoke | Bromundt (2013) | Light Therapy |
|  |  |  | Clark (2006) | Community Living Skills Course |
|  |  |  | Lee (2014) | Prevention and Recovery Centre (PARC) |
|  |  |  | Mehl (1997) | Art Therapy |
|  |  |  | Sayk (2025) | Imagery Rehearsal Therapy |
|  |  |  | Tomasi (2019) | Exercise and nutrition group |
| Recovery Assessment Scale – Domains and Stages (RAS-DS) | Yes | Hancock, N., Scanlan, J. N., Honey, A., Bundy, A. C., & O’Shea, K. (2015). Recovery assessment scale–domains and stages (RAS-DS): its feasibility and outcome measurement capacity. Australian & New Zealand Journal of Psychiatry, 49(7), 624-633. | Scanlan (2017) | Hospital to Home (H2H) |
| Risk characteristics | No | Not applicable/bespoke | Sekharan (2021) | Prevention and Recovery Care (PARC) |
| Rosenberg Self-Esteem Scale (RSES) | Yes | Rosenberg, M. (1965). Rosenberg self-esteem scale. Journal of Religion and Health. | de Girolamo (2024) | Structured physical activity intervention |
|  |  |  | Gabarda-Blasco (2024) | Adventure Therapy |
| Seasonal Pattern Assessment Questionnaire (SPAQ) | Yes | Rosenthal, N. E. (1984). Seasonal pattern assessment questionnaire. Journal of Affective Disorders. | Bromundt (2013) | Light Therapy |
| Service use (attendance and dropout) | No | Not applicable/bespoke | Soleimani (2025) | Coping Skills Training |
|  |  |  | Scanlan (2017) | Hospital to Home (H2H) |
| Service use (hospitalisation frequency) | No | Not applicable/bespoke | Brogan (2020) | Vital Mind Reset (VMR) online program |
|  |  |  | Lee (2014) | Prevention and Recovery Centre (PARC) |
|  |  |  | Sekharan (2021) | Prevention and Recovery Care (PARC) |
|  |  |  | Torres-Soto (2021) | Day Hospital treatment program |
| Service use (nursing records) | No | Not applicable/bespoke | Ortega (2004) | Functional behaviour analysis and treatment |
| Service use (treatment compliance) | No | Not applicable/bespoke | Bromundt (2013) | Light Therapy |
|  |  |  | Soleimani (2025) | Coping Skills Training |
| SIMple Physical Activity Questionnaire (SIMPAQ) | Yes | Rosenbaum, S., Morell, R., Abdel-Baki, A., Ahmadpanah, M., Anilkumar, T. V., Baie, L., ... & Ward, P. B. (2020). Assessing physical activity in people with mental illness: 23-country reliability and validity of the simple physical activity questionnaire (SIMPAQ). BMC psychiatry, 20(1), 108. | St-Amour (2024) | Structured physical exercise |
| Social Avoidance and Distress Scale (SAD) | Yes | Sobanski, J. A., Klasa, K., Rutkowski, K., Dembinska, E., Muldner-Nieckowski, L., & Cyranka, K. (2013). Social Avoidance and Distress Scale (SAD) and Fear of Negative Evaluation Scale (FNE)--reliability and the preliminary assessment of validity. Psychiatria polska, 47(4), 691-703. | Lima (2006) | Occupational Therapy |
| Sphigmomanometer (medical device, CE certified: 0476) | Yes | Standardised | de Girolamo (2024) | Structured physical activity intervention |
| State-Trait Anxiety Inventory (STAI) | Yes | Metzger, R. L. (1976). A reliability and validity study of the State-Trait Anxiety Inventory. Journal of Clinical Psychology, 32(2). | Bromundt (2013) | Light Therapy |
|  |  |  | de Girolamo (2024) | Structured physical activity intervention |
|  |  |  | Gabarda-Blasco (2024) | Adventure Therapy |
| State-Trait-Anger Inventory (STAXI) | Yes | Lievaart, M., Franken, I. H., & Hovens, J. E. (2016). Anger assessment in clinical and nonclinical populations: Further validation of the State–Trait Anger Expression Inventory‐2. Journal of clinical psychology, 72(3), 263-278. | Bromundt (2013) | Light Therapy |
| Symptom Checklist-90-Standard (SCL-90-S) | Yes | Franke, G. H. (2016, June). Titel Symptom-Checklist-90®-Standard 2. Zeile. In Diagnostische Verfahren in der Psychotherapie (Vol. 1, p. 426). Hogrefe Verlag GmbH & Company KG. | Sayk (2025) | Imagery Rehearsal Therapy |
| Symptom resolution | No | Not applicable/bespoke | Brogan (2020) | Vital Mind Reset (VMR) online program |
| Wide Range Achievement Test | Yes | Witt, J. C. (1986). Review of the wide range achievement test-revised. Journal of Psychoeducational Assessment, 4(1), 87-90. | Nelson (1987) | Vocational Readiness and Independent Living skills (VRIL) training program |
| Woodcock-Johnson Tests (Reading) | Yes | Abu-Hamour, B., Al Hmouz, H., Mattar, J., & Muhaidat, M. (2012). The use of Woodcock-Johnson tests for identifying students with special needs-a comprehensive literature review. Procedia-Social and Behavioral Sciences, 47, 665-673. | Nelson (1987) | Vocational Readiness and Independent Living skills (VRIL) training program |
| Work and Social Adjustment Scale (WSAS) | Yes | Lundqvist, J., Lindberg, M. S., Brattmyr, M., Havnen, A., Hjemdal, O., & Solem, S. (2024). The Work and Social Adjustment Scale (WSAS): An investigation of reliability, validity, and associations with clinical characteristics in psychiatric outpatients. PLoS One, 19(10), e0311420. | Scanlan (2017) | Hospital to Home (H2H) |
| Work hours | No | Not applicable/bespoke | Brogan (2020) | Vital Mind Reset (VMR) online program |
| Working Alliance Inventory (WAI) | Yes | Munder, T., Wilmers, F., Leonhart, R., Linster, H. W., & Barth, J. (2010). Working Alliance Inventory‐Short Revised (WAI‐SR): psychometric properties in outpatients and inpatients. Clinical Psychology & Psychotherapy: An International Journal of Theory & Practice, 17(3), 231-239. | St-Amour (2024) | Structured physical exercise |
| World Health Organization Disability Assessment Schedule (WHODAS 2.0) | Yes | Ciamarra, P., Corbi, G., Gimigliano, F., Feola, A., & Campobasso, C. P. (2025). The World Health Organization Disability Assessment Schedule 2.0 (WHODAS 2.0) as a measure among elderly population. A review. Disability and Rehabilitation, 47(15), 3773-3780. | de Girolamo (2024) | Structured physical activity intervention |
|  |  |  | Gabarda-Blasco (2024) | Adventure Therapy |
| World Health Organization Quality of Life Questionnaire - Abbreviated (WHOQOLBREF) | Yes | Aggarwal, A. N., Agarwal, R., & Gupta, D. (2014). Abbreviated World Health Organization Quality of Life questionnaire (WHOQOL-Bref) in north Indian patients with bronchial asthma: an evaluation using Rasch analysis. NPJ Primary Care Respiratory Medicine, 24(1), 1-6. | Gabarda-Blasco (2024) | Adventure Therapy |
| Wrist Actimetry (Actiwatch System) | Yes | Standardised | Bromundt (2013) | Light Therapy |
| Young Schema Questionnaire-Short Form (YSQ-SF) | Yes | Young, J. E. (1998). Young schema questionnaire--Short form. Cognitive therapy and research. | Soleimani (2025) | Coping Skills Training |
| Zanarini Rating Scale for BPD (ZAN-BPD) | Yes | Zanarini, M. C. (2003). Zanarini Rating Scale for Borderline Personality Disorder (ZAN-BPD): a continuous measure of DSM-IV borderline psychopathology. Journal of personality disorders, 17(3), 233-242. | de Girolamo (2024) | Structured physical activity intervention |
